# A modular vaccine platform enabled by decoration of bacterial outer membrane vesicles with biotinylated antigens

**DOI:** 10.1038/s41467-023-36101-2

**Published:** 2023-01-28

**Authors:** Kevin B. Weyant, Ayomide Oloyede, Sukumar Pal, Julie Liao, Mariela Rivera-De Jesus, Thapakorn Jaroentomeechai, Tyler D. Moeller, Steven Hoang-Phou, Sean F. Gilmore, Riya Singh, Daniel C. Pan, David Putnam, Christopher Locher, Luis M. de la Maza, Matthew A. Coleman, Matthew P. DeLisa

**Affiliations:** 1 Robert F. Smith School of Chemical and Biomolecular Engineering, Cornell University, Olin Hall, Ithaca, NY 14853 USA; 2 Nancy E. and Peter C. Meinig School of Biomedical Engineering, Cornell University, Weill Hall, Ithaca, NY 14853 USA; 3 Department of Pathology and Laboratory Medicine, Medical Sciences, Room D440, University of California, Irvine, Irvine, CA 92697 USA; 4 Versatope Therapeutics, Inc., 110 Canal Street, Lowell, MA 01852 USA; 5 Lawrence Livermore National Laboratory, Livermore, CA 94550 USA; 6 Cornell Institute of Biotechnology, Cornell University, Ithaca, NY 14853 USA

**Keywords:** Vaccines, Synthetic biology, Applied microbiology

## Abstract

Engineered outer membrane vesicles (OMVs) derived from Gram-negative bacteria are a promising technology for the creation of non-infectious, nanoparticle vaccines against diverse pathogens. However, antigen display on OMVs can be difficult to control and highly variable due to bottlenecks in protein expression and localization to the outer membrane of the host cell, especially for bulky and/or complex antigens. Here, we describe a universal approach for avidin-based vaccine antigen crosslinking (AvidVax) whereby biotinylated antigens are linked to the exterior of OMVs whose surfaces are remodeled with multiple copies of a synthetic antigen-binding protein (SNAP) comprised of an outer membrane scaffold protein fused to a biotin-binding protein. We show that SNAP-OMVs can be readily decorated with a molecularly diverse array of biotinylated subunit antigens, including globular and membrane proteins, glycans and glycoconjugates, haptens, lipids, and short peptides. When the resulting OMV formulations are injected in mice, strong antigen-specific antibody responses are observed that depend on the physical coupling between the antigen and SNAP-OMV delivery vehicle. Overall, these results demonstrate AvidVax as a modular platform that enables rapid and simplified assembly of antigen-studded OMVs for application as vaccines against pathogenic threats.

## Abstract (web summary)

Antigen display on outer membrane vesicles (OMVs) can be difficult to control and highly variable. Here, the authors describe a universal approach called AvidVax for linking biotinylated antigens to the exterior of OMVs and enabling rapid vaccine assembly.

## Introduction

Outer membrane vesicles (OMVs) are spherical bilayered nanostructures (~20–250 nm) ubiquitously released from the cell envelope of Gram-negative bacteria and their production represents a bona fide bacterial secretion process^1,2^. As derivatives of the cell envelope, OMVs mimic the structural organization and conformation of the bacterial cell surface while also containing periplasmic lumenal components. Natively produced OMVs mediate diverse functions such as increasing pathogenicity in the host environment^3^, promoting bacterial survival under conditions of stress^4^, and controlling interactions within microbial communities^5^.

In addition to their natural biological roles, OMVs have enabled a spectrum of bioengineering applications, most notably in drug and vaccine delivery, that exploit the unique structural and functional attributes of these nanoparticle systems^6–9^. OMVs are especially attractive as a vaccine platform because they are non-replicating, immunogenic facsimiles of the producing bacteria and thus contain the pathogen-associated molecular patterns (PAMPs) present on bacterial outer membranes^6,7^. These PAMPs endow OMVs with intrinsic immunostimulatory properties that strongly stimulate innate and adaptive immune response^10–13^. In addition to this in-built adjuvanticity, OMVs are right-sized for direct drainage into lymph nodes and subsequent uptake by antigen presenting cells and cross-presentation^14^. From a translational perspective, OMVs can be readily produced at high quantities and commercial scales via standard bacterial fermentation, and their clinical use has already been established in the context of OMVs from pathogenic *Neisseria meningitidis* serogroup B (MenB), also known as outer membrane protein complexes (OMPCs), that are the basis of a polyribosylribitol phosphate (PRP) conjugate vaccine approved for *Haemophilus influenzae* type b called PedvaxHIB®^15^ and are a component of the MenB vaccine Bexsero®^16^.

To generalize and expand the vaccine potential of OMVs, we and others have leveraged recombinant DNA technology and synthetic biology techniques to engineer OMVs with heterologous protein and peptide cargo^17,18^. By targeting expression to the outer membrane or the periplasm of an OMV-producing host strain, both surface display and lumenal encapsulation are possible, providing versatility as biomedical research tools and vaccines. Typically, this involves genetic fusion of a protein or peptide of interest (POI) to an outer membrane scaffold protein (e.g., the *E. coli* cytolysin ClyA), with the resulting POI accumulating in released OMVs that can be readily recovered from the culture supernatant. These methods have made it possible to enlist non-pathogenic, genetically tractable bacteria such as *Escherichia coli* K12 to produce designer OMVs that are loaded with foreign antigens of interest^6,19^. When inoculated in mice, such engineered OMVs stimulate antigen-specific humoral B cell and dendritic cell (DC)-mediated T-cell responses including activation of CD4^+^ and CD8^+^ T cells^10,20–22^. Importantly, the immune responses triggered by antigen-loaded OMV vaccines have proven to be protective against a range of foreign pathogens including bacteria and viruses^23–26^ as well as against malignant tumors^27^. While proteins and peptides remain the focus of most OMV-based vaccine efforts, advances in bacterial glycoengineering have enabled decoration of OMV exteriors with heterologous polysaccharide antigens, giving rise to an alternative type of glycoconjugate vaccines that can effectively deliver pathogen-mimetic glycan epitopes to the immune system and confer protection to subsequent pathogen challenge^28–30^. Collectively, these and other studies have revealed that the repetitive, high-density arrangement of antigens on the OMV surface enhances the response to otherwise poorly immunogenic epitopes such as small peptides and polysaccharides, which likely results from induction of strong B-cell receptor clustering.

These successes notwithstanding, the classical approach to loading OMVs with foreign antigens prior to their isolation from bacterial cultures is not without its challenges. For example, many antigens that are desirable from a vaccine standpoint are incompatible with recombinant expression in the lumen or on the surface of OMVs. While there can be many reasons for this, the most common bottlenecks include misfolding, proteolytic degradation, and/or inefficient bilayer translocation of the POI, especially for those that are very bulky and/or structurally complex. Because there are currently no effective tools for predicting a priori the expressibility of OMV-directed antigens, the creation of heterologous OMV vaccines remains very much a time-consuming trial-and-error process that often must be repeated for each new antigen. Even when a foreign antigen can be successfully localized to OMVs, it may lack important post-translational modifications that are formed inefficiently (or not at all) in the bacterial expression host. In addition, it can be difficult or even impossible to precisely control the quantity of OMV-associated antigen, thereby excluding antigen density as a customizable design parameter. It should also be noted that while it is possible to integrate polypeptide and polysaccharide biosynthesis with the vesiculation process^6,19^, it has yet to be demonstrated whether biosynthesis of other biomolecules can be similarly integrated, thereby limiting the spectrum of cargo that can be packaged in OMVs.

To address these shortcomings, reliable strategies are needed for modular OMV functionalization in which OMV vectors and structurally diverse target antigens are separately produced and then subsequently linked together in a controllable fashion. Along these lines, direct chemical conjugation of proteins and polysaccharides to OMVs/OMPCs following their isolation has been reported^15,31^; however, this technique involves non-specific attachment of antigens to unknown OMV components and thus is heterogeneous and difficult to predict or analyze. Moreover, non-uniform coupling of antigen to particulate carriers may result in sub-optimal immunogenicity. For more precise, homogenous antigen attachment, site-specific conjugation methods are preferable. To this end, specific bioconjugation on OMVs has been achieved by adapting a plug-and-display strategy that had previously been developed for decorating virus-like particles with protein and peptide antigens^32^. This approach involved the use of the SpyTag/SpyCatcher protein ligation system to covalently attach purified SpyTag-antigen (or SpyCatcher-antigen) fusion proteins onto cognate SpyCatcher-scaffold (or SpyTag-scaffold) fusions that were expressed on the surface of OMVs^33,34^. While this enabled loading of exogenous antigens on OMVs, with one report even demonstrating specific anti-tumor immune responses^33^, the protein ligation strategy is limited to antigens that are compatible with isopeptide bond formation. Furthermore, it has been shown that physical association of antigens with OMVs (as opposed to simple mixing of untethered antigens with OMVs) is critical to elicit strong immune responses against a variety of antigens including proteins, peptides, and polysaccharides^31,33,35^.

To develop a more universal strategy for tethering virtually any biomolecular cargo to the exterior of OMVs, we created a method for avidin-based vaccine antigen crosslinking (AvidVax) whereby biotinylated antigens are linked to the exterior of OMVs whose surfaces are remodeled with biotin-binding proteins. The method involves producing OMV vectors that display multiple copies of a synthetic antigen-binding protein (SNAP) comprised of an outer membrane scaffold protein fused to a member of the avidin family. Following their production and isolation, SNAP-OMVs can be readily decorated with a wide range of biotinylated subunit antigens, including globular and membrane proteins, glycans and glycoconjugates, haptens, lipids, and short peptides. Importantly, antigen-studded SNAP-OMVs promote strong antigen-specific antibody responses that compare favorably to the responses measured for classically prepared OMV formulations (i.e., cellular expression of antigen-scaffold fusions). Overall, our results demonstrate that AvidVax is a highly modular and versatile platform for vaccine creation that should enable rapid cycles of development, testing, and production of antigen-studded OMVs for use as vaccines against pathogenic agents.

## Results

### A modular framework for specific attachment of antigens on OMV surfaces

**Fig. 1:**
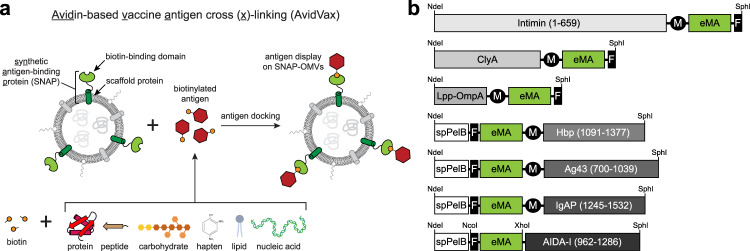
A modular platform for rapid self-assembly of OMV-based vaccine candidates. **a** Schematic of AvidVax technology whereby ready-made OMVs displaying a synthetic antigen receptor (SNAP-OMVs) are remodeled with biotinylated antigens-of-interest. Using AvidVax, the surface of SNAP-OMVs can be remodeled with virtually any biomolecule that is amenable to biotinylation including peptides, proteins, carbohydrates, glycolipids, glycoproteins, haptens, lipids, and nucleic acids. Schematic created with BioRender.com. **b** Genetic architecture of SNAP constructs tested in this study. Numbers in parentheses denote amino acids of the scaffold that were fused to the biotin-binding eMA domain and used for membrane anchoring. Additional features include: export signal peptide from PelB (spPelB); c-Myc epitope tag (M); FLAG epitope tag (F), and NdeI, Xhol, SphI, and NcoI restriction enzyme sites used for cloning.

As a first step towards developing a universal platform for rapidly assembling antigens of interest on the surface of OMVs, we constructed SNAPs by fusing a cell surface scaffold protein to a biotin-binding protein (Fig. 1a). A panel of cell surface scaffold modules were chosen based on their ability to direct passenger proteins to the *E. coli* outer membrane. These included cytolysin ClyA^18^, hybrid protein Lpp-OmpA^36^, and the autotransporter β-domains derived from the N-terminus of intimin (Int)^37^ and the C-termini of adhesin involved in diffuse adherence (AIDA-I), antigen-43 (Ag43), hemoglobin-binding protease (Hbp), and immunoglobulin A protease (IgAP)^38^. Initially, each scaffold was fused in-frame to enhanced monoavidin (eMA) (Fig. 1b), a derivative of dimeric rhizavidin (RA) that was designed to be monomeric with highly stable, biotin-binding properties^39^, and subsequently expressed from the arabinose-inducible plasmid pBAD24 in hypervesiculating *E. coli* strain KPM404 Δ*nlpI*. This strain is an endotoxin-free derivative of BL21(DE3)^40^ (sold as ClearColi^TM^ by Lucigen) that we previously engineered to hypervesiculate through knockout of the *nlpI* gene^41^. Using this strain, OMVs were produced that contained full-length SNAP chimeras, with Lpp-OmpA-eMA and Int-eMA showing the strongest expression albeit with significant amounts of higher and lower molecular weight species that likely corresponded to aggregation and degradation products, respectively (Supplementary Fig. 1a).

To evaluate antigen docking, we initially focused on biotinylated green fluorescent protein (biotin-GFP) as the target antigen (Supplementary Fig. 2a), which enabled facile and quantitative prototyping of the different SNAP-OMV designs. When biotin-GFP was incubated with 100 ng SNAP-OMVs immobilized on ELISA plates, all exhibited dose-dependent binding up to ~10 nM of biotin-GFP except for the eMA-AIDA-Iβ and ClyA-eMA receptors, which appeared saturated at low levels of biotin-GFP (Supplementary Fig. 1b). The lack of binding for these two SNAPs was not entirely surprising given that these constructs exhibited weak expression compared to the other SNAPs (Supplementary Fig. 1b). Importantly, there was no detectable binding of unmodified GFP by any of the SNAP-OMVs, indicating that antigen capture was entirely dependent upon the presence of the biotin moiety. Next, the two most effective SNAPs in terms of biotin-GFP binding, eMA-IgAPβ and Lpp-OmpA-eMA, were evaluated over a range of conditions to identify parameters (e.g., growth temperature, culture density at time of induction, inducer level, etc.) that affected GFP docking levels (Supplementary Note and Supplementary Fig. 3). Overall, the engineered Lpp-OmpA-eMA receptor outperformed eMA-IgAPβ in terms of biotin-GFP binding capacity (Supplementary Fig. 3a); however, expression of this construct from pBAD24 using standard amounts of L-arabinose (0.2% or 13.3 mM) was detrimental to the host cells based on the observation that the final culture densities hardly changed, and in some cases even decreased, from the densities at the time of induction, which was not the case for eMA-IgAPβ (Supplementary Fig. 3b). Given the different biogenesis pathways of the IgAP autotransporter versus the Lpp-OmpA β-barrel outer membrane protein, we suspected that the host cell toxicity associated with Lpp-OmpA might result from inducer levels that were too strong. In support of this notion, when Lpp-OmpA-eMA constructs were induced with ~50-times less inducer, the post-induction cell growth was markedly improved, with Lpp-OmpA-eMA-expressing cells reaching a final density on par with that of cells expressing eMA-IgAPβ (Supplementary Fig. 3c). Importantly, the Lpp-OmpA-eMA SNAP-OMVs isolated from these healthier host cells captured more biotin-GFP compared to eMA-IgAPβ SNAP-OMVs. An even higher level of biotin-GFP binding was obtained by moving the Lpp-OmpA-eMA construct into the L-rhamnose-inducible plasmid pTrham (Supplementary Fig. 4), which is known for its tighter expression control compared to pBAD vectors and can help to overcome the deleterious saturation of membrane and secretory protein biogenesis pathways^42,43^.

To determine the effect of the biotin-binding module on antigen loading and to further highlight the modularity of AvidVax, we constructed a panel of Lpp-OmpA-based SNAPs comprised of alternative biotin-binding domains including dimeric RA, tetrameric streptavidin (SA), and monomeric streptavidin with a lowered off-rate (mSA^S25H^)^44^. The SNAPs comprised of RA and mSA^S25H^ both captured biotin-GFP at a level that was nearly identical to the eMA-based receptor, while the SA-based receptor showed binding that was barely above background, a result that appears to be due to the poor expression of this SNAP compared to the others (Supplementary Fig. 5a, b). Given the similarity in antigen capture efficiency for the eMA, RA, and mSA^S25H^ SNAPs as well as post-induction culture growth (Supplementary Fig. 5a), we chose the more extensively characterized Lpp-OmpA-eMA SNAP (expressed from plasmid pTrham with 0.5 mM L-rhamnose inducer) for all further studies. Importantly, we observed virtually no batch-to-batch variation in the expression and stability of this construct in OMV fractions corresponding to three independently prepared batches, regardless of whether samples were analyzed immediately or subjected to a freeze-thaw cycle (Supplementary Fig. 6a).

### SNAP-OMVs enable controllable antigen loading density

**Fig. 2:**
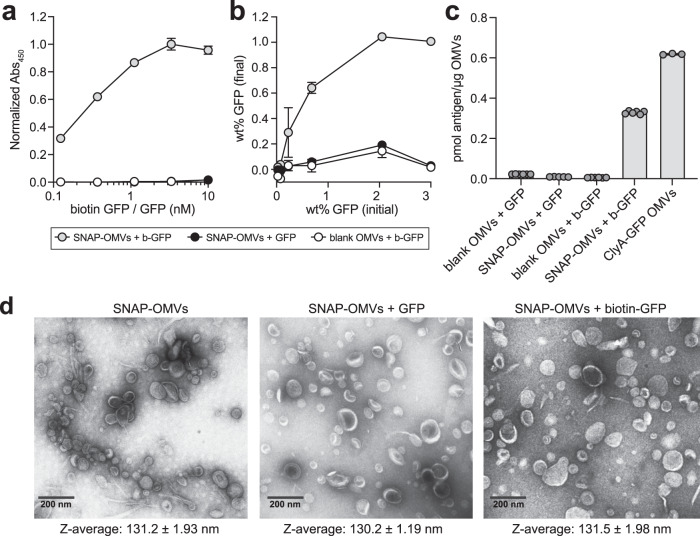
Chimeric Lpp-OmpA-eMA SNAP enables controllable antigen loading on OMVs. **a** Dose-response curve generated by loading biotin-GFP (b-GFP) or unmodified GFP (GFP) on SNAP-OMVs isolated from hypervesiculating *E. coli* strain KPM404 Δ*nlpI* expressing the Lpp-OmpA-eMA construct from plasmid pTrham (induced with 0.5 mM L-rhamnose). Blank OMVs were isolated from plasmid-free KPM404 Δ*nlpI* cells. Binding activity was determined by ELISA in which Lpp-OmpA-eMA SNAP-OMVs were immobilized on plates and subjected to varying amounts of biotin-GFP, after which plates were extensively washed prior to detection of bound biotin-GFP using anti-polyhistidine antibody to detect C-terminal 6xHis tag on GFP. Data were normalized to the maximum binding signal corresponding to Lpp-OmpA-eMA SNAP-OMVs in the presence of 3.3 nM biotin-GFP. Data are the mean ± SD with *n* = 2 biologically independent experiments. **b** Same OMVs as in (**a**) but dose-response was generated by first incubating OMVs with biotin-GFP or unmodified GFP in solution, washing to remove unbound protein, and determining GFP levels by ELISA-based detection using a standard curve with known amounts of GFP mixed with OMVs. Data are the mean ± SD with *n* = 3 biologically independent experiments. **c** Comparison of GFP levels on Lpp-OmpA-eMA SNAP-OMVs versus ClyA-GFP OMVs. ClyA-GFP OMVs were isolated from KPM404 Δ*nlpI* cells expressing ClyA-GFP fusion construct from plasmid pBAD18 as described in Kim et al.^18^. Data are the mean ± SD with *n* = 6 biologically independent experiments for all cases except for ClyA-GFP OMVs where *n* = 3 biologically independent experiments. **d** Transmission electron micrograph of Lpp-OmpA-eMA SNAP-OMVs alone or following incubation with unmodified GFP or biotin-GFP as indicated. The scale bar represents 200 nm. The Z-average diameter of each formulation was measured by DLS and reported below each corresponding micrograph. Micrographs representative of two independently repeated experiments with similar results. Source data are provided as a Source Data file.

To determine the loading capacity of Lpp-OmpA-eMA SNAP-OMVs, the OMV fractions were first subjected to extensive washing with ultracentrifugation to recover washed OMVs, and then irreversible aggregates were removed by filtration through 0.45 μm pores. Next, we quantified the amount of bound antigen by mixing Lpp-OmpA-eMA SNAP-OMVs with biotin-GFP in solution, washing OMVs by ultracentrifugation to remove soluble protein, and subsequently measuring the amount of OMV-bound GFP by coating the sample on an ELISA plate and comparing the anti-GFP signal to standards with known amounts of GFP mixed with OMVs. This assay was designed to be used for any antigen and mirror the process of vaccine assembly, whereby ready-made SNAP-OMVs are mixed with biotinylated antigens in an on-demand fashion. Importantly, the dose-response profile when mixing biotin-GFP and SNAP-OMVs in solution was in relative agreement with the dose-response curve generated by capturing biotin-GFP on the surface of pre-immobilized SNAP-OMVs (Fig. 2a) and showed good batch-to-batch consistency (Supplementary Fig. 6a). The maximum amount of biotin-GFP that was captured on the SNAP-OMV surface was ~1% by mass (i.e., 0.01 μg biotin-GFP/μg OMV) when ~2 wt % biotin-GFP was input to the mixture, with the addition of higher amounts of biotin-GFP leading to no significant increase in biotin-GFP binding (Fig. 2b). In both assay formats, the combination of SNAP-OMVs with unbiotinylated GFP or biotin-GFP with blank OMVs lacking a SNAP resulted in little to no detectable binding (Fig. 2a, b). We also found that the maximum biotin-GFP loading on SNAP-OMVs on a molar basis was lower but on par with the amount of GFP that was displayed on the surface of OMVs following cellular expression of a scaffold-antigen fusion, ClyA-GFP (Fig. 2c)^18^. Despite this difference, an advantage of SNAP-OMVs was the ability to vary the antigen loading density over a wide biotin-GFP concentration range, thereby providing a level of control that is more difficult to achieve with cellular expression of scaffold-antigen fusions. When visualized by transmission electron microscopy (TEM), SNAP-OMVs decorated with biotin-GFP had a size (~50 nm) and overall appearance that was indistinguishable from unloaded SNAP-OMVs (Fig. 2d) and consistent with previous TEM images of engineered OMVs^18,22,25^ including those from the same hypervesiculating host strain used here^41^. Furthermore, there was no evidence of aggregation for SNAP-OMVs decorated with biotin-GFP as determined by measuring the Z-average diameter of OMV formulations by dynamic light scattering (DLS) (Fig. 2d and Supplementary Fig. 6b). These findings indicate that controllable vesicle loading could be achieved using the AvidVax approach without significantly impacting OMV ultrastructure.

### SNAP-OMVs enable specific attachment of diverse antigen molecules

**Fig. 3:**
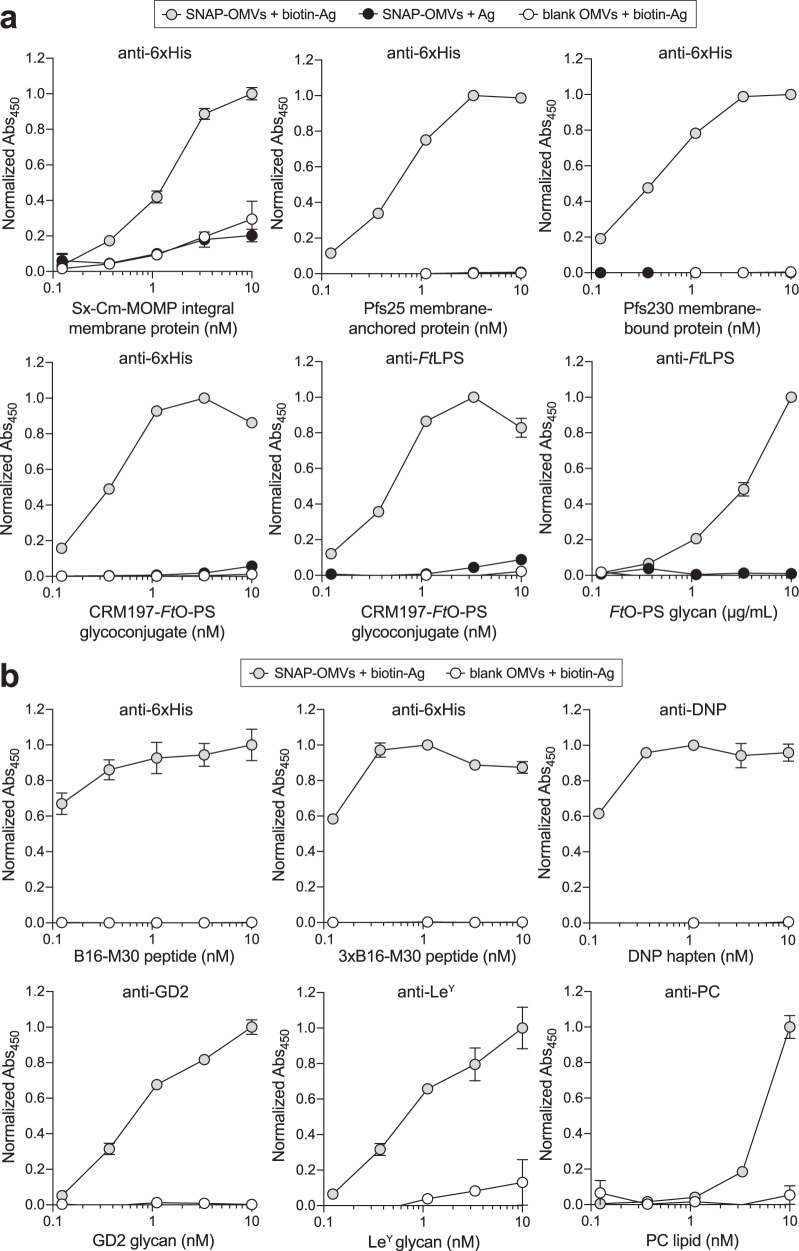
Assembly of OMV vaccine candidates decorated with diverse biomolecular antigens. **a**, **b** Dose–response curves generated by loading biotinylated or unbiotinylated antigens on SNAP-OMVs isolated from hypervesiculating KPM404 Δ*nlpI* cells expressing the Lpp-OmpA-eMA construct from plasmid pTrham (induced with 0.5 mM L-rhamnose). Blank OMVs were isolated from plasmid-free KPM404 Δ*nlpI* cells. Binding activity was determined by ELISA in which Lpp-OmpA-eMA SNAP-OMVs were immobilized on plates and subjected to varying amounts of unbiotinylated or biotinylated antigen, after which plates were extensively washed prior to detection of bound antigen using the antibodies indicated at top of each panel. Data were normalized to the maximum binding signal in each experiment. Data are the mean ± SD with *n* = 2 biologically independent experiments. Source data are provided as a Source Data file.

To demonstrate the universality of the approach, we next investigated decoration of SNAP-OMVs with a structurally diverse array of biotinylated antigens. Some of these were chosen because their incorporation into the OMV structure through cellular expression as a scaffold-antigen fusion protein was predicted to be difficult or impossible. For example, *Chlamydia* major outer membrane protein (MOMP) is a β-barrel integral membrane protein (IMP) that accounts for ~60% of the mass of the outer membrane of *Chlamydia* spp.^45,46^ and is highly antigenic^47^, making it an attractive subunit vaccine candidate^48^. However, expression of MOMP in the *E. coli* cytoplasm results in aggregation and the formation of inclusion bodies^49,50^ while expression in the *E. coli* outer membrane results in significant cell toxicity^49,51,52^. Along similar lines, *Plasmodium falciparum* Pfs25 protein (Pfs25), a glycophosphotidylinositol (GPI)-anchored protein expressed on the surface of zygotes and ookinetes, and Pfs230, a 230-kDa membrane-bound gamete protein, are promising malaria transmission-blocking vaccine antigens^53,54^ but both proteins are very difficult to express recombinantly in *E. coli*^54,55^. To render these three challenging membrane protein antigens compatible with OMVs required generation of soluble versions of each antigen. For MOMP from *Chlamydia trachomatis* mouse pneumonitis (MoPn) biovar (strain Nigg II; now called *Chlamydia muridarum*), soluble expression was achieved using a protein engineering technology known as SIMPLEx (solubilization of IMPs with high levels of expression)^56^ in which sandwich fusion between an N-terminal decoy protein, namely *E. coli* maltose-binding protein (MBP), and C-terminal truncated human apolipoprotein AI (ApoAI*) transformed *C. muridarum* MOMP (Cm-MOMP) into a water-soluble protein that was expressed at high levels in the *E. coli* cytoplasm (Supplementary Fig. 2a). For Pfs25 and Pfs230, soluble expression was achieved using a baculovirus-insect cell expression system (Supplementary Fig. 2b, c). Following incubation of SNAP-OMVs with biotinylated versions of *E. coli*-derived SIMPLEx-Cm-MOMP (Sx-Cm-MOMP) and insect cell-derived Pfs25 and Pfs230, we observed efficient OMV decoration that depended on both the presence of the chimeric Lpp-OmpA-eMA receptor on OMVs and the biotin moiety on each antigen (Fig. 3a). In the case of Sx-Cm-MOMP, we observed a low but reproducible signal for both controls (SNAP-OMVs with unbiotinylated Sx-Cm-MOMP and blank OMVs with biotinylated Sx- Cm-MOMP) that may correspond to a small amount of auto-insertion of Sx-Cm-MOMP into OMVs. Furthermore, we determined that ~0.2–0.3 pmol antigen / μg SNAP-OMVs of biotinylated Sx-Cm-MOMP and Pfs230 was tethered specifically, which was on par with the amount of captured biotin-GFP on a molar basis (Fig. 2c).

We next investigated carbohydrate structures such as lipopolysaccharide (LPS) antigens that represent appealing molecules for vaccine development owing to their ubiquitous presence on the surface of diverse pathogens and malignant cells. A challenge faced with most polysaccharides is that they make poor vaccines on their own because they are unable to interact with the receptors on T cells in germinal centers (GCs)^57^. This can be overcome by covalent attachment of a polysaccharide to a carrier protein, which provides T-cell epitopes that can induce polysaccharide-specific IgM-to-IgG class switching, initiate the process of affinity maturation, and establish long-lived memory^58^. Despite the widespread success of glycoconjugates, there is an unmet need to identify formulations that elicit stronger primary antibody responses after a single immunization, especially in primed or pre-exposed adolescents and adults, and achieve prolonged vaccine efficiency^58^. To this end, we speculated that AvidVax would provide a convenient strategy for combining glycoconjugates with the intrinsic adjuvant properties of OMVs^10–12^. Such an approach would provide a simpler alternative than attempting to combine OMV biogenesis with cellular expression of glycoconjugate vaccine candidates in *E. coli*^59^, a feat that has yet to be reported. Thus, we attempted to adorn SNAP-OMVs with biotinylated glycoconjugates by leveraging an engineered *E. coli* strain^60^ to produce the carrier protein CRM197 that was glycosylated at its C-terminus with a recombinant mimic of the *Francisella tularensis* SchuS4 O-antigen polysaccharide (*Ft*O-PS) (Supplementary Fig. 2d). Decoration of SNAP-OMVs with a biotinylated version of this glycoconjugate was readily detected by immunoblotting against both the CRM197 carrier and its covalently linked *Ft*O-PS antigen (Fig. 3a). We also demonstrated an alternative strategy for combining OMVs with polysaccharide antigens whereby a biotinylated version of *F. tularensis* SchuS4 LPS (*Ft*LPS) was directly bound to the exterior of SNAP-OMVs (Fig. 3a). This formulation was motivated by the fact that a protein providing T-cell help only needs to be in close proximity to the polysaccharide in order to target the same B cell and does not have to be covalently linked to the polysaccharide to induce class switching and T-cell activation^28,29,61^. Indeed, the co-delivery of non-covalently linked proteins and polysaccharides present on the exterior of OMVs is sufficient to make a polysaccharide immunogenic^15,28,29^.

The final group of antigens that we investigated were small-sized biomolecules that are known to be weakly immunogenic by themselves and therefore require carrier molecules to increase chemical stability and adjuvanticity for the induction of a robust immune response. This group included: (i) B16-M30 peptide, a CD4^+^ T‐cell neoepitope expressed in the B16F10 melanoma as a consequence of a mutation in the *kif18b* gene^62^, and a longer variation that contained three tandem repeats of the B16-M30 epitope (3xB16-M30); (ii) ganglioside GD2 glycan, a pentasaccharide antigen found on human tumors including melanoma, neuroblastoma, osteosarcoma, and small-cell lung cancer, that was highly ranked (12 out of 75) in a National Cancer Institute pilot program that prioritized the most important cancer antigens^63^; (iii) Lewis Y (Le^Y^), a tetrasaccharide extension of the H blood group galactose-glucosamine that has been shown to be overexpressed on tumors^64^; (iv) 2,4-dinitrophenol (DNP), a model hapten to which the immune system is unresponsive^65^; and (v) phosphocholine (PC), a major lipid component of myelin and one of the main antigenic targets of the autoimmune response in multiple sclerosis, with lipid-reactive antibodies likely contributing to disease pathogenesis^66^. In each case, we observed clearly detectable antigen binding on the surface of SNAP-OMVs that was significantly above the background seen with blank OMVs lacking biotin-binding receptors (Fig. 3b). Collectively, these results illustrate the potential of the AvidVax approach for modular self-assembly of candidate OMV vaccines decorated with diverse biomolecular cargo.

### SNAP-OMVs amplify the immunogenicity of physically associated GFP

**Fig. 4:**
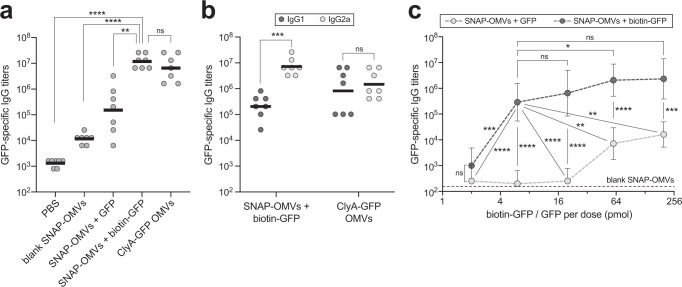
SNAP-OMVs decorated with biotin-GFP boost GFP-specific IgG titers. **a** Mean GFP-specific IgG titers (horizontal black lines) in endpoint (day 56) serum of individual mice in each group (gray circles). Five groups of six-week-old BALB/c mice (seven mice per group) were immunized s.c. with the following: PBS, blank SNAP-OMVs, SNAP-OMVs mixed with 20 pmol unbiotinylated or biotinylated GFP, and ClyA-GFP OMVs displaying a ClyA-GFP fusion. All OMVs were isolated from KPM404 Δ*nlpI* cells with the appropriate expression plasmid. Mice received prime injections containing an equivalent amount of OMVs (20 μg total protein) on day 0 and were boosted on day 21 and 42 with the same doses. **b** Mean GFP-specific IgG1 and IgG2a titers in the same endpoint serum of individual mice in select groups from (**a**). **c** Mean GFP-specific IgG titers in mice receiving SNAP-OMVs formulated with escalating doses (2–200 pmol) of biotinylated GFP (physically associated, dark gray circles) or non-biotinylated GFP (non-associated mixtures, light gray circles). An equivalent amount of SNAP-OMVs was used in each case. Dashed black line indicates mean GFP-specific IgG titer of blank SNAP-OMV control group. Data are the mean titers of 6 mice in each group ± SD. Statistical significance was determined by two-tailed *t* test with Welch’s correction (*p < 0.05; **p < 0.01; ***p < 0.001; ****p < 0.0001; ns not significant). Actual *p* values in (**a**) from left-to-right: *p* < 0.0001, *p* < 0.0001, *p* = 0.0013, and *p* = 0.2751. Actual *p* values in (**b**) from left to right: *p* < 0.0001  and *p* = 0.5184 . Actual *p* values in (**c**) from left-to-right: *p* = 0.1401, *p* = 0.0001, *p* < 0.0001, *p* < 0.0001, *p* = 0.4719, *p* < 0.0001, *p* < 0.0001, *p* = 0.0413, *p* = 0.0022, *p* = 0.0072, *p* = 0.0654, *p* < 0.0001, *p* = 0.0004. Source data are provided as a Source Data file.

We next sought to assess the immunological potential of SNAP-OMVs decorated with GFP as a model antigen. Specifically, BALB/c mice were immunized via subcutaneous (s.c.) injection of SNAP-OMVs specifically attached to biotin-GFP after which blood was collected at regular intervals. Negative control groups included PBS, blank SNAP-OMVs, and SNAP-OMVs that were mixed with unbiotinylated GFP. ClyA-GFP-containing OMVs generated by cellular expression, which were previously reported to elicit high antibody titers following immunization in mice^22^, served as a positive control. Importantly, SNAP-OMVs displaying biotin-GFP elicited robust IgG responses to GFP that were significantly higher than the titers measured for control mice immunized with blank SNAP-OMVs or PBS (Fig. 4a). It is particularly noteworthy that the total IgG titers triggered by SNAP-OMVs were indistinguishable from those measured in response to ClyA-GFP-containing OMVs, validating the antigen docking strategy as a potent alternative to cellular expression of scaffold-antigen fusions for boosting the immunogenicity of foreign subunit antigens, in particular those that are weakly immunogenic on their own such as GFP^22,67^.

To determine whether the immune responses were Th1 or Th2 biased^68^, IgG antibody titers were further broken down by analyzing IgG1 and IgG2a subclasses. Mice immunized with different GFP-containing OMVs showed robust mean titers of both GFP-specific IgG1 and IgG2a antibodies (Fig. 4b). For the groups immunized with ClyA-GFP OMVs, the relative titers of IgG1 and IgG2a subclasses were comparable, which was entirely consistent with the subclass distributions seen previously for identically prepared OMV formulations^41^. In contrast, biotin-GFP-studded SNAP-OMVs elicited an IgG2a-dominant humoral response, suggesting induction of a Th1-biased immune response consistent with heightened cellular immunity stimulation. While the reasons for this Th1 bias remain unclear, it does not appear to result from different LPS levels because the amount of endotoxin measured in the two formulations, SNAP-OMVs with biotin-GFP and ClyA-GFP OMVs, was indistinguishable as determined using a standard bacterial endotoxin test (Supplementary Fig. 6c). Other possible factors that we cannot rule out are different immunostimulatory properties of the protein antigens themselves (biotin-GFP versus GFP) or the overexpressed scaffold proteins used to anchor them to OMVs (Lpp-OmpA versus ClyA).

Interestingly, the IgG response elicited by non-tethered GFP that was mixed with SNAP-OMVs gave a significantly lower antigen-specific IgG response compared to biotin-GFP that was docked on OMVs (Fig. 4a), suggesting that physical coupling of antigens to the OMV surface is essential for exploiting the full intrinsic adjuvanticity of OMVs. To investigate this phenomenon more deeply, we evaluated simple mixing of antigen with OMVs versus physical association of antigens on OMVs in a systematic, quantitative manner. For this experiment, an equivalent amount of SNAP-OMVs was incubated with escalating doses of either biotinylated or non-biotinylated GFP over a 100-fold range (2–200 pmol, where the middle 20 pmol dose reflects the standard amount tested above) and the humoral responses to the resulting formulations were measured. We observed that the GFP-specific serum IgG titers were significantly greater for the SNAP-OMVs loaded with biotinylated GFP versus those that were simply mixed with an equivalent amount of non-biotinylated GFP (Fig. 4c). This trend was observed at each of the GFP doses tested except for the lowest, with neither of the 2 pmol-formulations giving a significant signal above the blank SNAP-OMV control group.

Importantly, SNAP-OMVs that were stably associated with as little as 6 pmol (0.18 μg) of biotinylated GFP antigen elicited IgG titers that were significantly higher than the responses measured for any of the formulations involving SNAP-OMVs mixed with non-biotinylated GFP that was not specifically attached (Fig. 4c). Moreover, the 6 pmol dose of stably associated biotin-GFP was sufficient to reach the plateau of antigen-specific antibody titers that was similarly reached with the highest dose tested (200 pmol of stably associated biotin-GFP). In contrast, none of the non-associated formulations reached this plateau. In fact, only the 60 and 200 pmol doses of non-biotinylated GFP mixed with SNAP-OMVs were capable of eliciting IgG titers that were significantly above the blank SNAP-OMV control group, and yet the IgG titers at these relatively high doses were still ~2 logs lower than the corresponding responses to GFP that was physically associated with SNAP-OMVs. It is worth noting that the maximum amount of biotinylated GFP that can be bound specifically to the amount of SNAP-OMVs used here (20 μg of total protein) is ~6.7 pmol based on our quantitative analysis (Fig. 2a–c). While it remains unclear why formulations with >6 pmol of biotinylated GFP did not provide much benefit, we speculate that either ~6 pmol of antigen was sufficient to reach a saturating antibody response or that formulations containing greater amounts of GFP resulted in excess antigen that was not specifically bound to OMVs.

### SNAP-OMVs elicit IgG antibodies to linear peptide epitopes

Previous studies demonstrated that tandemly repeated linear peptides recombinantly expressed as fusions to OMV scaffold proteins can induce peptide-specific antibodies^25,27^. To determine if the SNAP-OMV platform could also induce humoral immune responses to short, linear peptides, we turned our attention to the B16-M30 peptide (PSKPSFQEFVDWENVSPELNSTDQPFL). This peptide is best known as a CD4^+^ T‐cell neoepitope^62^ that can elicit M30-specific, CD4+ T cells following immunization with OMVs that were mixed with the B16-M30 peptide^27^. However, bioinformatic analysis using the linear B-cell epitope prediction tool BepiPred 2.0^69^ revealed a putative B-cell epitope spanning residues Q7 to Q24 (Supplementary Fig. 7a). To determine the immunogenicity of this sequence, and at the same time extend our method to linear peptide antigens, we immunized BALB/c mice with SNAP-OMVs that were physically associated with a biotinylated peptide that contained three tandem repeats of the B16-M30 epitope. Immunization of mice with SNAP-OMVs in which biotinylated 3xB16-M30 was specifically associated resulted in significant peptide-specific IgG responses that were on par with the responses observed in mice immunized with OMVs displaying an Lpp-OmpA-3xB16-M30 fusion protein that was generated by cellular expression (Supplementary Fig. 7b). The latter construct was expressed at a level that was indistinguishable from the Lpp-OmpA-eMA SNAP fusion, suggesting that nearly equivalent quantities of 3xB16-M30 were displayed in these formulations (Supplementary Fig. 7c). These results demonstrate the ability of SNAP-OMVs to elicit antibodies against short, linear peptides containing B-cell epitopes that can be quickly and easily synthesized and docked onto the surface of ready-made SNAP-OMVs.

### SNAP-OMVs elicit neutralizing antibodies against membrane protein antigens

**Fig. 5:**
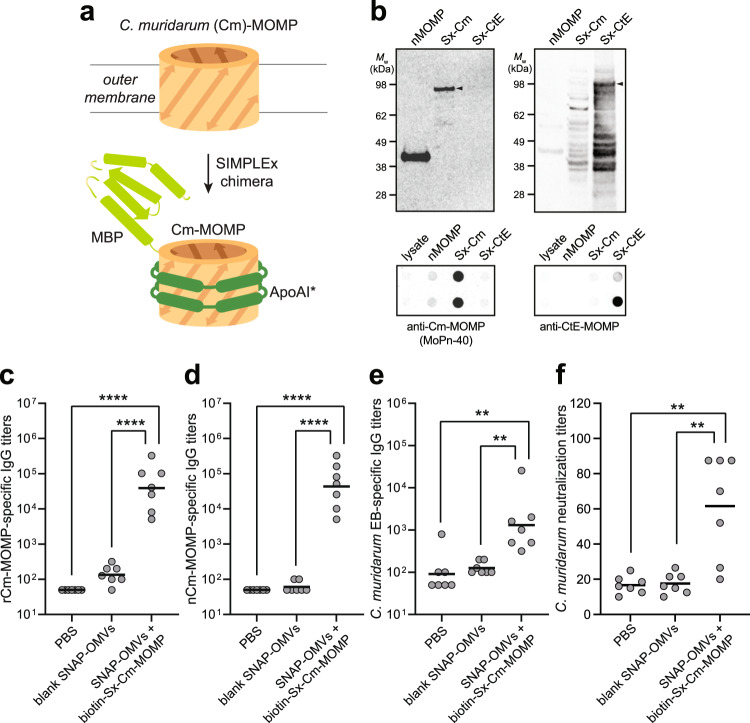
SNAP-OMVs decorated with biotinylated Sx-Cm-MOMP elicit neutralizing IgGs. **a** Schematic of SIMPLEx strategy for converting integral membrane proteins into water-soluble proteins that can be expressed at high titers in the cytoplasm of host cells. Here, the β-barrel outer membrane protein Cm-MOMP was fused at its N-terminus with *E. coli* maltose-binding protein (MBP) and at its C-terminus with truncated ApoAI (ApoAI*). Structural analysis indicates that ApoAI* adopts a belt-like conformation around the membrane helices of proteins to which it is fused, effectively shielding these highly hydrophobic segments from water^56^. Schematic created with BioRender.com. (**b**, left blot) Antigenicity of Sx-Cm-MOMP construct evaluated by immunoblot analysis using mAb MoPn-40. Native Cm-MOMP (nCm-MOMP) and Sx-CtE-MOMP served as positive and negative controls, respectively. (**b**, right blot) The latter construct was detected with commercial antibody specific for CtE-MOMP, which did not react with Sx-Cm-MOMP or nMOMP. Expected location of full-length SIMPLEx fusion proteins are denoted by black arrows. Molecular weight (*M*_w_) ladder is indicated at left. Blots representative of two independently repeated experiments with similar results. **c** Antigen-specific IgG titers against recombinant preparations of Cm-MOMP (rCm-MOMP; horizontal black lines) in endpoint (day 56) serum of individual mice in each group (gray circles). Three groups of six-week-old BALB/c mice (seven mice per group) were immunized s.c. with the following: PBS, blank SNAP-OMVs, and SNAP-OMVs mixed with biotinylated Sx-Cm-MOMP. Mice received prime injections containing an equivalent amount of OMVs (20 μg total protein) on day 0 and were boosted on day 21 and 42 with the same doses. **d**, **e** Same as in (**c**) but with either (**d**) a native preparation of Cm-MOMP (nCm-MOMP) or (**e**) elementary bodies (EBs) as immobilized antigens. **f** In vitro neutralizing titers in endpoint (day 56) serum of individual mice. Each dot corresponds to an individual mouse and horizontal line indicates mean titer. Statistical significance was determined by two-tailed *t* test with Welch’s correction (**p < 0.01, ****p < 0.0001). Actual *p* values: **c** *p* < 0.0001 and *p* < 0.0001; **d** *p* < 0.0001 and *p* < 0.0001; **e** *p* = 0.0024 and *p* = 0.0051; and **f** *p* = 0.0064 and *p* = 0.0069. Source data are provided as a Source Data file.

Encouraged by the immunostimulatory effects of SNAP-OMVs decorated with model protein and peptide antigens, we next investigated the humoral immune response to SNAP-OMVs that were specifically associated with Cm-MOMP, a validated subunit vaccine candidate^48,50^. Prior to immunization, we first tested the antigenicity of our Sx-Cm-MOMP construct (Fig. 5a) that was engineered as described above for soluble, high-level expression. Immunoblots of purified Sx-Cm-MOMP were probed with anti-Cm-MOMP-specific monoclonal antibody (mAb) MoPn-40, which was generated by inoculation of BALB/c mice with *C. muridarum* followed by isolation of hybridomas producing antibodies against Cm-MOMP^70^. We observed that mAb MoPn-40 specifically recognized the water-soluble Sx-Cm-MOMP construct but not a SIMPLEx control construct comprised of a different MOMP from *C. trachomatis* serovar E (Sx-CtE-MOMP) in both denatured immunoblots and non-denatured dot blots (Fig. 5b), indicating that water-soluble Sx-Cm-MOMP retained conformational antigenicity.

Next, BALB/c mice were immunized s.c. with SNAP-OMVs decorated with biotinylated Sx-Cm-MOMP and their sera were tested for IgG reactivity against preparations of recombinant and native Cm-MOMP (rCm-MOMP and nCm-MOMP, respectively)^50^ as well as intact *C. muridarum*, and for functional antibody activity by an in vitro neutralization assay. The SNAP-OMVs displaying biotinylated Sx-Cm-MOMP induced strong IgG responses to both antigens with total IgG titers that were significantly greater than the titers elicited by blank SNAP-OMVs and PBS control groups (Fig. 5c, d). The IgG responses triggered by Sx-Cm-MOMP docked on SNAP-OMVs were confirmed to be *Chlamydia-*specific as evidenced by binding to *C. muridarum* elementary bodies (EBs), with binding that was significantly above the binding measured for blank SNAP-OMVs and PBS control groups (Fig. 5e). Most importantly, these IgG responses correlated with an enhanced ability to neutralize *C. muridarum* infectivity. Specifically, we found that SNAP-OMVs decorated with biotinylated Sx-Cm-MOMP elicited high *C. muridarum*-specific neutralizing titers that were significantly above control groups, demonstrating the ability of AvidVax vaccine candidates to elicit functional antibodies (Fig. 5f). Furthermore, these results confirm that dock-and-display of SIMPLEx-solubilized membrane protein variants on SNAP-OMVs is a unique approach for rapidly engineering vaccines based on difficult-to-obtain membrane-bound protein antigens without compromising antigenicity or immunogenicity.

## Discussion

In this study, we have developed a universal platform called AvidVax for rapidly assembling antigens of interest on the surface of OMVs. The method involves site-specific docking of biotinylated antigens to the exterior of ready-made OMVs displaying multiple copies of highly modular receptors called SNAPs, which are engineered by fusing an outer membrane scaffold domain to a biotin-binding domain. As we showed here, SNAP-OMVs can be readily adorned with virtually any antigen that is amenable to biotinylation including globular and membrane proteins, glycans and glycoconjugates, haptens, lipids, and short peptides. The ability to precisely and homogenously load OMVs with a diverse array of subunit antigen molecules differentiates the AvidVax method from previous covalent conjugation strategies that are largely restricted to protein and peptide antigens^31,33,34^. Moreover, our dock-and-display approach side-steps many of the challenges associated with display on OMVs using conventional genetic fusion and cellular expression technology, thereby opening the door to important vaccine subunit antigens such as *Chlamydia* Cm-MOMP and malarial Pfs25 and Pfs230 that are refractory to soluble expression and outer membrane localization in *E. coli*^49–52,55^. While the separate preparation of a biotinylated antigen adds an extra step, it affords an opportunity to generate protein antigens using different expression systems, which can be chosen based on their ability to promote high yields and desired conformations including post-translational modifications.

When injected in wild-type BALB/c mice, SNAP-OMV formulations displaying GFP or a water-soluble variant of Cm-MOMP triggered strong antigen-specific humoral responses that depended on the physical linkage between the antigen and the SNAP-OMV delivery vehicle. Importantly, the antigen-specific IgG titers elicited by SNAP-OMVs with biotinylated GFP that was physically attached rivaled those of ClyA-GFP-containing OMVs generated by conventional cellular expression technology^22^. This ability of SNAP-OMVs to amplify the immunogenicity of GFP, a weakly immunogenic protein by itself^22,67^, without the need for potentially reactogenic adjuvants indicates that the inbuilt adjuvanticity of OMVs is preserved in the context of our dock-and-display strategy. Moreover, the IgG responses to SNAP-OMVs with stably associated GFP were far superior to those induced by formulations involving simple mixtures of OMVs and GFP that was not specifically associated. In fact, physical association to the OMV surface enabled doses as small as 6 pmol (0.18 μg) to elicit plateau antigen-specific IgG responses, whereas this same plateau could not be reached from formulations involving non-associated GFP even at doses that contained up to 33 times more antigen. Together, these results justify the extra effort associated with antigen biotinylation.

In the case of the validated subunit vaccine candidate, Cm-MOMP^48,50^, we demonstrate the potential of AvidVax to be readily combined with SIMPLEx, a technology for solubilizing integral membrane proteins^56,71^, leading to an effective strategy for formulating difficult-to-obtain antigens without compromising immunogenicity. Future adaptations of AvidVax could also be pursued as needed such as increasing antigen density with tandemly repeated biotin-binding modules or enabling multi-antigen display with SNAPs comprised of multiple orthogonal protein-ligand binding pairs. Along these lines, we previously engineered a trivalent protein scaffold containing three divergent cohesin domains for the position-specific docking of a three-enzyme cascade on the exterior of OMVs^72^, which provides a conceptual starting point for next-generation SNAP-OMVs.

The AvidVax technology is based on the extraordinarily high affinity of avidin for the small molecule biotin and was found to be compatible with a range of different biotin-binding modules including eMA, RA, and mSA^S25H^. Although the binding affinity of our preferred biotin-binding domain, eMA, toward free biotin is measurably weaker than tetrameric SA (*K*_d_ = 31 × 10^−12^ M for eMA versus ~10^−14^ M for SA), eMA is reported to have almost multimeric avidin-like binding stability toward biotin conjugates^39^, making it an incredibly useful module for capturing diverse subunit antigens as we showed here. Moreover, its small, monomeric design resulted in significantly better expression and OMV localization of the Lpp-OmpA-eMA SNAP compared to Lpp-OmpA-SA, which in turn resulted in far superior antigen capture. Another notable trait of eMA is its ability to be stored at −20 °C without visible aggregation or loss of binding function^39^, which could prove useful in the future for long-term vaccine storage. It should be noted that the versatility of the avidin-biotin technology has been previously leveraged as a building material in other types of vaccine formulations, enabling the attachment of antigens onto virus-like particles (VLPs)^73–75^ and the self-assembly of macromolecular complexes comprised of vaccine antigens^76,77^. However, to our knowledge, our study is the first to repurpose avidin-biotin for antigen assembly and display on OMVs.

Overall, the AvidVax platform enables creation of antigen-studded OMVs with the potential to impact many important facets of vaccine development. For example, the simplicity and modularity of vaccine assembly using AvidVax enables rapid cycles of development and testing, which could be useful for evaluating large numbers and different combinations of pathogen-derived antigens for their ability to combat the most intractable diseases such as malaria or tuberculosis. Moreover, the fact that AvidVax is based on an identical, easy-to-decorate SNAP-OMV scaffold that can be readily mass produced could shorten the time from development to manufacturing and accelerate regulatory review for each new vaccine candidate. The universal scaffold also affords the ability to share production costs across multiple antigens and diseases, which in combination with the favorable manufacturing economics of *E. coli-*based production, could help to lower the cost per vaccine dose. In addition, pre-production of modular OMV scaffolds that can be stably stored at −20 °C and then only need to be mixed with good manufacturing practice (GMP)-grade biotinylated antigens could enable rapid responses to pathogen outbreaks or pandemics. Nonetheless, significant enhancements in SNAP-OMV yields will need to be achieved as we obtained only 1–2 mg SNAP-OMV/liter culture using the methods described in this work. Furthermore, one major remaining obstacle is that OMVs derived from laboratory strains of *E. coli* have yet to enter the clinic. It should be noted, however, that OMVs/OMPCs from *Neisseria meningitidis* serogroup B are the basis of two licensed vaccines, PedvaxHIB® and Bexsero®, that are approved for use in humans^15,16^. Hence, although more testing of SNAP-OMV vaccine candidates is clearly required, including broader immunogenicity testing and pathogen challenge studies, we anticipate that clinical translation may not be far off.

## Methods

### Strains, growth media, and plasmids

All OMVs in this study were isolated from the hypervesiculating *E. coli* strain KPM404 Δ*nlpI*^41^, which was created by introducing several genetic modifications to the K12 strain BL21(DE3) that rendered its LPS less reactogenic^40^. *E. coli* strain BL21(DE3) (Novagen) was used to express GFP and rCm-MOMP. The SIMPLEx constructs Sx-Cm-MOMP and Sx-CtE-MOMP were produced in two different ways, cell-based expression with BL21(DE3) or cell-free expression using an *E. coli*-based translation kit (RTS 500 ProteoMaster *E. coli* HY kit, Biotechrabbit GmbH) as per the manufacturer’s protocol. Both methods yield comparable amounts of similar quality products as assessed by SDS-PAGE and immunoblot analysis. *E. coli* strain CLM24 was used to produce CRM197 conjugated with *Ft*O-PS^60^ while strain JC8031^78^ was used to produce recombinant *Ft*LPS. Recombinant GFP used for serum antibody titering was expressed and purified from *Saccharomyces cerevisiae* strain SEY6210.1 to avoid cross-reaction of serum antibodies with contaminating host proteins present in protein preparations derived from *E. coli* cultures. The *C. muridarum* strain Nigg II (ATCC VR-123) was used to produce nCm-MOMP and EBs utilized in serum antibody titering experiments.

For cloning and strain propagation, *E. coli* strains were grown on solid Luria-Bertani LB (10 g/L tryptone, 10 g/L NaCl, and 5 g/L yeast extract) supplemented with agar (LBA) and yeast strain SEY6210.1 was grown on synthetic defined media without uracil (SD-URA; MP Biomedicals) supplemented with agar. For OMV production, hypervesiculating *E. coli* were grown in terrific broth (TB) (12 g/L tryptone, 24 g/L yeast extract, 0.4% v/v glycerol, 0.17 M KH_2_PO_4_ and 0.72 M K_2_HPO_4_). For production of recombinant antigens using *E. coli*, cells were grown in LB media. SEY6210.1 was grown in SD-URA or yeast extract-peptone-dextrose (YPD) media (20 g/L peptone, 10 g/L yeast extract and 2% w/v glucose). *C. muridarum* cells were grown according to standard protocols^79,80^.

All plasmids used in this study are described in Supplementary Table 1. For plasmid construction, DNA encoding all target genes was synthesized as gBlocks (Integrated DNA Technologies, IDT) and cloned into plasmids using standard restriction enzyme-based cloning methods. All plasmid DNA sequences were confirmed through Sanger sequencing performed by the Cornell Biotechnology Resource Center (BRC) unless specified otherwise. For expression of SNAP constructs in OMVs, eMA fusions to ClyA, Lpp-OmpA, and the membrane-associated transporter domains of the autotransporters Int, Hbp, Ag43, and IgAP were codon-optimized for *E. coli* expression, synthesized, and cloned into plasmid pBAD24^81^ between EcoRI and SphI restriction sites with an NdeI site at the start codon by GenScript. SNAPs involving ClyA, Lpp-OmpA and Int were cloned with eMA fused to the 3′ end of the scaffold while SNAPs involving Hbp, Ag43 and IgAP were cloned with eMA fused to the 5′ end of the scaffold (Fig. 1b). For the latter set of constructs, DNA encoding a Sec-dependent export signal peptide derived from PelB (spPelB), identical to the sequence in pET22b (Novagen), was introduced at the 5′-end of the eMA-scaffold gene fusions. For all of these constructs, DNA encoding c-Myc (EQKLISEEDL) and FLAG (DYKDDDDK) epitope tags was introduced at the 5′ and 3′ ends of eMA as depicted in Fig. 1b. In the case of the autotransporter AIDA-I, the transporter unit (amino acids 962 through 1286) was PCR-amplified from pIB264^82^ and ligated into pBAD24 between XhoI and SphI restriction sites. A gBlock (IDT) encoding eMA with a 5′ FLAG tag (IDT) was ligated between NcoI and XhoI restriction sites, after which a gBlock encoding spPelB (IDT) was ligated between EcoRI and NcoI.

To construct L-rhamnose inducible plasmids, DNA encoding Lpp-OmpA-eMA and eMA-IgAP was digested from the respective pBAD24 expression vectors and ligated into pTrham (Amid Biosciences) between NdeI and SphI sites, yielding plasmids pTrham-Lpp-OmpA-eMA and pTrham-eMA-IgAP, respectively. To construct SNAPs based on alternative avidin domains, *Rhizobium etli* RA, *Streptomyces avidinii* SA, and an optimized version of monomeric streptavidin, namely mSA^S25H^, with a lowered off-rate^44^ were codon-optimized and synthesized as gBlocks with flanking BbsI and HindIII restriction sites (IDT). The sequences were then used to replace the eMA sequence in the pTrham-Lpp-OmpA-eMA vector, resulting in plasmids pTrham-Lpp-OmpA-RA, pTrham-Lpp-OmpA-SA, and pTrham-Lpp-OmpA-mSA.

For expression of GFP antigen for docking on OMVs, the gene encoding FACS-optimized GFPmut2 with a C-terminal 6xHis tag was cloned in pET24a(+)-Cm^R^ between SacI and HindIII restriction sites, yielding pET24-GFP. For yeast expression of GFP used in serum antibody titering, a codon-optimized gene encoding GFPmut2 was synthesized as a double-stranded DNA fragment or gBlock (IDT) with a 5′ Kozak sequence and 3′ 6xHis tag and ligated into the yeast-expression plasmid pCM189 (ATCC) between BamHI and PstI sites, yielding pCM-GFP. For expression of the Sx-Cm-MOMP antigen for OMV docking studies, the sequence encoding codon-optimized Cm-MOMP^83^ was synthesized as a gBlock (IDT) and ligated into the SIMPLEx plasmid pET21d-Sx^71^ between NdeI and EcoRI restriction sites, yielding pET21-Sx-Cm-MOMP. A modified strategy was used to generate plasmid pIVEX-Sx-CtE-MOMP encoding the Sx-CtE-MOMP construct. Briefly, PCR products corresponding to codon-optimized CtE-MOMP^83^, MBP, and ApoAI* (human ApoAI with 49 N-terminal amino acids removed) were cloned into pIVEX-2.4d using the following restriction enzyme strategy: NdeI-MBP-XhoI-MOMP-NsiI-ApoA1-SacI. The plasmid sequence was confirmed through Sanger sequencing performed by Elim Biopharm.

### Protein purification

For production of GFP and Sx-Cm-MOMP protein antigens, BL21(DE3) cells containing plasmids corresponding to each antigen were grown overnight at 37 °C in 5 mL LB supplemented with the appropriate antibiotic and subcultured 1:100 into the same media. Protein expression was induced with 0.1 mM isopropyl-β-D-1-thiogalactopyranoside (IPTG) when culture densities reached an absorbance at 600 nm (Abs_600_) of ~1.0 and proceeded for 16 h at 30 °C. Cells were then harvested and lysed by homogenization, and proteins were purified by Ni-NTA resin (Thermo-Fisher) following the manufacturer’s protocol. For Sx-Cm-MOMP, Ni-NTA resin elute was immediately diluted with PBS containing 1 mM EDTA (PBS-E) and incubated with amylose resin (New England Biolabs) for 30 min, followed by washing with 10 resin volumes of PBS-E and elution with 10 mM maltose in PBS-E. All purified proteins were buffer exchanged into PBS using PD-10 desalting columns (Cytiva), filter-sterilized, quantified by Lowry (MilliporeSigma), and stored at 4 °C for up to 2 months or at −80 °C for longer term storage. Pfs25 and Pfs230 were expressed and purified from a baculovirus expression system using *Spodoptera frugiperda* Sf9 cells and P2 virus by Syngene.

To produce CRM197-*Ft*O-PS glycoconjugate, *E. coli* strain CLM24 was transformed with plasmid pTrc99S-spDsbA-CRM197^4xDQNAT^ encoding the CRM197 carrier protein modified at its C-terminus with four tandemly repeated DQNAT glycosylation motifs^84^, plasmid pGAB2 encoding the *Ft*O-PS biosynthesis pathway^60^, and plasmid pMAF10-PglB encoding the *Campylobacter jejuni* oligosaccharyltransferase PglB for transfer of the *Ft*O-PS^85^. Overnight cultures were subcultured 1:100 into fresh LB containing appropriate antibiotics. When culture densities reached Abs_600_ of ~0.8, PglB expression was induced with 0.2% arabinose for 16 h at 30 °C, at which point CRM197^4xDQNAT^ expression was induced with 0.1 mM IPTG and cells were grown for an additional 8 h at 30 °C. Cells were then harvested and purified as described above for GFP.

To purify GFP for serum antibody titering, yeast strain SEY6210.1 was transformed with pCM189-GFP-6xHis and grown on SD-URA agar plates at 30 °C for two days. Afterwards, a colony was picked and grown overnight at 30 °C in 5 mL of SD-URA media containing tetracycline, subcultured 1:10 into YPD, and grown for 20 h at 30 °C. Yeast cells were lysed by homogenization and protein was purified by Ni-NTA resin as above. For Cm-MOMP serum antibody titering, rCm-MOMP was expressed recombinantly in *E. coli* while nCm-MOMP was extracted from *C. muridarum* strain Nigg II.

### Antigen biotinylation

Purified GFP, Sx-Cm-MOMP, Pfs25, Pfs230, and CRM197-*Ft*O-PS were mixed at 1 mg/mL (0.25-1 mg total protein) with 1.5x molar excess EZ-Link Sulfo-NHS-LC biotin (Thermo-Fisher) in PBS and incubated on ice for 2-3 h. Afterwards, the reaction mix was passed five times over PBS-equilibrated monomeric avidin resin (Thermo-Fisher). Final flow-through fractions were concentrated and saved for repeat biotinylation reactions as needed. Following 6 washes each with one resin volume of PBS, biotinylated protein was eluted 6 times each with one resin volume of 2 mM D-biotin (MilliporeSigma) in PBS. Elutions were pooled and diluted to 6 mL with PBS and concentrated to <200 μL using 6-mL, 10-kDa cut-off protein concentrators (Pierce). Dilution and concentration was repeated three more times to remove the d-biotin. The final concentrated biotinylated proteins were filter-sterilized, quantified by Lowry, and stored at 4 °C for up to 2 months.

To biotinylate *F. tularensis* LPS, an aliquot of in-house produced *Ft*LPS^28^ was buffer exchanged into PBS using PD-10 columns to remove sugar monomers and short polysaccharide chains and then quantified by the Purpald assay^86^. Biotinylation was performed using 1-cyano-4-dimethylaminopyridinium tetrafluoroborate (CDAP) as the activation reagent linking EZ-Link-Amine-PEG3-Biotin (Pierce) to hydroxyl groups on the polysaccharide^76^. A 27-amino acid B16-M30 peptide with N-terminal biotin and C-terminal polyhistidine (6xHis) motif for antibody-based detection was synthesized by Biomatik to ~85% purity, and a 1 mg/mL stock was prepared in dimethyl sulfoxide (DMSO). A longer peptide with the B16-M30 peptide sequence tandemly repeated three times (3xB16-M30) was similarly prepared by Biomatik. Biotinylated GD2 ganglioside oligosaccharide and biotinylated Le^Y^ oligosaccharide were purchased from Elicityl, biotinylated DNP containing a polyethylene glycol (PEG) linker was purchased from Nanocs, and 1-oleoyl-2-[12-biotinyl(aminododecanoyl)]-sn-glycero-3-phosphocholine (18:1-12:0 biotin-PC) powder was purchased from Avanti Polar Lipids. The biotin-GD2, biotin-Le^Y^, and biotin-DNP were dissolved in sterile water (1–5 mg/mL) while biotin-PC was suspended in DMSO (1 mg/mL). All stocks were diluted in PBS for avidin binding studies.

### OMV preparation

KPM404 Δ*nlpI* cells containing pBAD24 or pTrham expression plasmids were spread from -80 °C glycerol stocks onto LBA plates supplemented with 100 μg/ml carbenicillin and grown overnight at 37 °C (~20 h). On the following day, cells were suspended from the agar using TB and subcultured to Abs_600_ of ~0.06 in 50–100 mL TB supplemented with carbenicillin. Cells were grown at 37 °C and 220 rpm and induced when Abs_600_ reached ~0.6 to ~1.8 with varying concentrations of l-arabinose (pBAD24) or l-rhamnose (pTrham). Following induction, cells were grown for 16 h at 28 °C followed by 6 h at 37 °C, after which cells were pelleted via centrifugation at 10,000 × *g* for 15 min. Supernatants were filtered through 0.2 μm filters and stored overnight at 4 °C. OMVs were isolated by ultracentrifugation at 141,000 × *g* for 3 h at 4 °C and resuspended in sterile PBS. For quantitative analysis and immunizations, resuspended OMVs were diluted in sterile PBS and ultracentrifuged a second time to remove residual media and soluble proteins. Following a second resuspension in PBS, large irreversible aggregates were removed by centrifuging for 2 min at 3000 × *g* in a microcentrifuge and filtering the supernatant using sterile 4-mm, 0.45-μm syringe filters (MilliporeSigma). Total OMV proteins were quantified by Lowry (Peterson’s modification; MilliporeSigma) using bovine serum albumin (BSA) as protein standard. OMVs were stored for up to 1 month at 4 °C for binding analysis and up to 2 weeks prior to immunizations. Bacterial endotoxin testing was performed on OMV fractions using the Limulus amebocyte lysate (LAL) assay kit (GenScript L00350Y) according to manufacturer’s protocol. Endotoxin concentration in OMV formulations was quantified based on a standard curve generated using *E. coli* LPS^22^.

### Immunoblot analysis

Biotinylated and unbiotinylated protein antigens and OMVs were mixed with loading buffer containing β-mercaptoethanol and boiled for 10 min prior to loading onto Mini-PROTEAN TGX polyacrylamide gels (Bio-Rad). To determine protein purity, gels were stained with Coomassie G-250 stain (Bio-Rad) following the manufacturer’s protocol. For immunoblot analysis, proteins were transferred to polyvinylidene difluoride (PVDF) membranes and blocked with 5% milk followed by probing with antibodies, which were all used at 1:5,000 dilution. Avidin expression on OMVs was analyzed with horseradish peroxidase (HRP)-conjugated anti-c-Myc (Abcam, Cat # ab19312) or HRP-conjugated anti-DDDDK (Abcam, Cat # ab1162) antibodies that recognized c-Myc and FLAG epitope tags, respectively. Proteins and peptides bearing C-terminal 6xHis tags were detected with mouse anti-6xHis antibody clone AD1.1.10 (BioRad, Cat # MCA1396GA) while detection of glycosylated CRM197-*Ft*O-PS was with anti-*F. tularensis* LPS antibody clone FB11 (Invitrogen, Cat # MA1-21690) that is specific to *Ft*LPS^28^. HRP-conjugated goat anti-mouse (Abcam, Cat # ab6789) was used as needed. All membranes were developed using Clarity ECL substrate (Bio-Rad) and visualized using a ChemiDoc imaging system (Bio-Rad). Image Lab 6.1 software (Bio-Rad) was used for collecting Western blot images.

For probing antigenicity of SIMPLEx constructs, Sx-Cm-MOMP and Sx-CtE-MOMP were mixed with loading buffer containing DTT and boiled for 10 min before loading onto 4–12% NuPAGE Bis-Tris gels (Thermo-Fisher). For denaturing immunoblot analysis, proteins were transferred to PVDF membranes and blocked with 5% BSA (Sigma) followed by probing with mAb MoPn-40 (1:1000 dilution)^70^ or anti-CtE-MOMP (1:2000 dilution; Novus Biologicals, Cat # NB100-66403) antibodies. For non-denaturing dot blot analysis, purified Sx-Cm-MOMP and Sx-CtE-MOMP proteins were spotted directly onto nitrocellulose membranes and incubated for 5 min before being blocked with 5% BSA (Sigma) and probing with the same primary antibodies. IRDye 800CW-conjugated goat anti-mouse secondary antibodies (1:10,000 dilution; Li-Cor, Cat # 926-32210) were used to detect primary antibody binding and membranes were visualized using an Odyssey Li-Cor Fc imaging system.

### TEM and DLS analysis of OMVs

To characterize OMV formulations, antigens and OMVs were mixed in PBS to a final concentration of 100 pmol/mL and 0.1 mg/mL, respectively, for at least 1 h at room temperature. For TEM analysis, OMVs were negatively stained with 1.5% uranyl acetate and deposited on 300-mesh Formvar carbon-coated copper grids. Imaging was performed using a FEI Tecnai 12 BioTwin transmission electron microscope. For DLS size analysis, Z-averages of OMV formulations were measured in triplicate with the refractive index set to 1.395 using a Nano ZS (Malvern Instruments) and Dynamic V6 software.

### ELISA

For qualitative assessment of antigen binding by SNAP-OMVs, OMV samples were diluted to 2 μg/mL in PBS, coated on Costar 9018 high-binding 96-well plates (50 μL per well), and incubated overnight at 4 °C. Plates were blocked with 2% BSA in PBS (100 μL/well) for 3 h at room temperature and subsequently washed two times with PBST (PBS pH 7.4 with 0.005% Tween-20 and 0.3% BSA). To analyze relative binding capacities, biotinylated or unbiotinylated antigens were serially diluted in triplicate by a factor of 3 in PBST, starting from 10 nM, and incubated for 90 min at room temperature (50 μL/well). Unbound antigen was removed by washing twice with PBST. Bound antigen was labeled by incubating with primary antibody for 1 h in PBST followed by two more PBST washes and a 1-h incubation with HRP-conjugated secondary antibody. After three final washes with PBST, 3,3′-5,5′-tetramethylbenzidine substrate (1-Step Ultra TMB-ELISA; Thermo-Fisher) was added and the plate was incubated at room temperature for 30 min in the dark. The reaction was stopped with 2 M H_2_SO_4_ and absorbance was measured via microplate spectrophotometer (Molecular Devices) at Abs_450_. The absorbance reading from OMVs incubated with PBST without antigen was subtracted from the signal in all wells with antigen added. The resulting values were normalized to the highest average absorbance value among all antigen concentrations, including unbiotinylated antigen controls. Primary anti-6xHis and anti-*Ft*LPS antibodies and HRP-conjugated anti-mouse secondary antibody were identical to those used for immunoblotting above and were used at the same dilutions. The remaining antigens were detected with the following antibodies: GD2 was detected with mouse anti-ganglioside GD2 antibody (1:1000 dilution; Abcam, Cat # ab68456); Le^Y^ was detected with mouse anti-Lewis Y antibody clone H18A (1:1000 dilution; Absolute Antibody, Cat # Ab00493-1.1); DNP was detected with goat anti-DNP (1:5,000 dilution; Bethyl Laboratories, Cat # A150-117A); and PC was detected with anti-phosphorylcholine antibody clone BH8 (1:250 dilution; MilliporeSigma, Cat # MABF2084). HRP-conjugated donkey anti-goat secondary was used as needed (1:5000 dilution; Abcam, Cat # ab97110).

For quantification of antigen-binding capacity on SNAP-OMVs, 50 μg OMVs were diluted to 0.1 mg/mL in PBS, mixed with unbiotinylated or biotinylated GFP at concentrations between 0 and 100 pmol/mL (0–1 pmol antigen/μg OMV), and incubated at room temperature for 1 h. Mixtures were then diluted to 30 mL in PBS and ultracentrifuged for 141,000 × *g* for 3 h at 4 °C. After discarding the supernatant, the pellet was resuspended with 100 μL PBS, and the washed OMVs were quantified by Lowry (Peterson’s modification). An ELISA-based method that could be applied to a variety of molecules was then used to quantify the amount of antigen remaining. Specifically, washed OMVs were diluted to 2 μg/mL and coated on high-binding ELISA plates (Costar 9018) in triplicate (50 μL per well). Known standards were prepared by mixing blank SNAP-OMVs at 2 μg/mL with 1:2 serial dilutions of unbiotinylated or biotinylated GFP, starting from 1 pmol GFP/μg OMV, and coating each antigen concentration in triplicate on the ELISA plates (50 μL per well). Following overnight coating at 4 °C, plates were blocked with 2% BSA in PBS for 3 h (100 μL per well) at room temperature and subsequently washed two times with PBST. Antibody and substrate incubations were identical to the qualitative binding ELISA described above. The final Abs_450_ signals of the OMV mixtures containing known amounts of unbiotinylated or biotinylated antigen were used to generate standard curves from which the amount of antigen remaining in each unknown washed OMV sample was calculated. The amount of GFP displayed on positive-control ClyA-GFP OMVs was quantified via fluorescence microplate reader (Molecular Devices).

### Mouse immunizations

One day prior to immunization (day -1), different OMV formulations were diluted to 0.1 mg/mL in sterile PBS pH 7.4. For the formulations involving docked antigens, antigens and OMVs were mixed to a final concentration of 100 pmol/mL and 0.1 mg/mL, respectively, corresponding to 1 pmol antigen/μg OMV (~3 wt% for GFP). All formulations were immediately stored at 4 °C. On day 0, 200 μL (20 μg) OMVs were injected s.c. into six-week-old BALB/c mice (seven mice per group). Mice were housed under the following environmental conditions to reduce stress: 14-hour light/10-hour dark cycle at ~70 °F with ~50% humidity. Identical booster injections were administered on days 21 and 42, and blood was drawn from the mandibular sinus on days -1, 28 and 49. Mice were euthanized on day 56, which was immediately followed by blood collection via cardiac puncture and spleen collection. The protocol number for the animal trial was 2012-0132 and was approved by the Institutional Animal Care and Use Committee (IACUC) at Cornell University.

### Serum antibody titers

Sera was isolated from the blood of immunized mice by centrifugation at 5000 × *g* for 10 min and stored at −20 °C. Antigen-specific antibodies in the sera were measured using indirect ELISA. Briefly, high-binding 96-well plates (Costar 9018) were coated with purified antigen (5 μg/mL in PBS, pH 7.4) and incubated overnight at 4 °C, followed by overnight blocking with 5% non-fat dry milk (Carnation) in PBS. Serum samples were serially diluted in triplicate by a factor of two in blocking buffer, starting from 1:100, and incubated on the antigen-coated plates for 2 h at 37 °C. Plates were washed 3 times with PBST and incubated for 1 h at 37 °C in the presence of one of the following HRP-conjugated antibodies: goat anti-mouse IgG (1:10,000; Abcam Cat # ab6789); anti-mouse IgG1 (1:10,000; Abcam Cat # ab97240), or anti-mouse IgG2a (1:10,000; Abcam Cat # ab97245). Following 3 final washes with PBST, 1-Step Ultra TMB-ELISA (Thermo-Fisher) was added and the plate was incubated at room temperature for 30 min in the dark. The reaction was stopped with 2 M H_2_SO_4_, and absorbance was quantified via microplate spectrophotometer (Molecular Devices) at Abs_450_. Serum antibody titers were determined by measuring the highest dilution that resulted in signal three standard deviations above no-serum background controls.

The *Chlamydia*-specific antibody titers in sera from mice immunized with Sx-Cm-MOMP were determined by ELISA. Briefly, 96-well plates were coated with 2 μg/ml of rCm-MOMP or nCm-MOMP, or 100 μL/well of *C. muridarum* EBs containing 10 μg/mL of protein in PBS. Next, 100 μL of serum was added per well in serial dilutions. Following incubation at 37 °C for 1 h, the plates were washed, and HRP-conjugated goat anti-mouse IgG (1:10,000; BD Biosciences Cat # 554002) was added. Binding was measured in a plate reader (Labsystem Multiscan) using 2,2′-azino-bis-(3-ethylbenzthiazoline-6-sulfonate) as substrate.

### *C. muridarum* neutralization titers

For in vitro neutralization, assays were performed in 96-well plates with two-fold serial dilutions of mouse sera in Ca^2+^- and Mg^2+^-free PBS (pH 7.2) supplemented with 5% guinea pig serum were incubated with *C. muridarum* EBs (1 × 10^4^ infectious units (IFU)) for 45 min at 37 °C. The mixtures were added to HeLa-229 cell monolayers and centrifuged for 1 h and incubated at 37 °C for 30 h. After washing, the monolayers were fixed with methanol and stained with an in-house monoclonal antibody 40 recognizing variable domain 1 of the major outer membrane protein of *C. muridarum*^70^. The titer of a sample was the dilution that yielded 50% neutralization relative to the negative control serum from PBS-immunized mice.

### Statistical analysis and reproducibility

To ensure robust reproducibility of all results, experiments were performed with at least three biological replicates and at least three technical measurements. Sample sizes were not predetermined based on statistical methods but were chosen according to the standards of the field (at least three independent biological replicates for each condition), which gave sufficient statistics for the effect sizes of interest. All data were reported as geometric mean with error bars representing standard deviation (SD). Statistical significance was determined by Welch’s *t*-test and *p*-values of <0.05 were considered significant. All graphs were generated using GraphPad Prism 9 for MacOS version 9.4.1. No data were excluded from the analyses. The experiments were not randomized. The Investigators were not blinded to allocation during experiments and outcome assessment.

### Reporting summary

Further information on research design is available in the Nature Portfolio Reporting Summary linked to this article.

## Supplementary information

Supplementary Information

Reporting Summary

## Data Availability

All data generated or analyzed during this study are included in this article and its Supplementary Information/Source Data file. Source data are provided with this paper.

## Source data

Source Data
