# Supplementary material for: A modular vaccine platform enabled by decoration of bacterial outer membrane vesicles with biotinylated antigens
Source: Nat Commun. 2023 Jan 28;14:464. doi: 10.1038/s41467-023-36101-2 (PMC9883832; doi:10.1038/s41467-023-36101-2)
Supplement: Supplementary file 1 — Supplementary Information [file 41467_2023_36101_MOESM1_ESM.pdf]

Supplementary Information

A modular vaccine platform enabled by decoration of bacterial outer membrane vesicles with biotinylated antigens

Kevin B. Weyant[1], Ayomide Oloyede[2], Sukumar Pal[3], Julie Liao[4], Mariela Rivera-De Jesus[2], Thapakorn Jaroentomeechai[1], Tyler D. Moeller[1], Steven Hoang-Phou[5], Sean F. Gilmore[5], Riya Singh[1], Daniel C. Pan[4], David Putnam[1,2], Christopher Locher[4], Luis M. de la Maza[3], Matthew A. Coleman[5] and Matthew P. DeLisa[1,2,6*]

[1]Robert F. Smith School of Chemical and Biomolecular Engineering, Cornell University, Olin Hall, Ithaca, NY 14853 USA
[2]Nancy E. and Peter C. Meinig School of Biomedical Engineering, Cornell University, Weill Hall, Ithaca, NY 14853 USA
[3]Department of Pathology and Laboratory Medicine, Medical Sciences, Room D440, University of California, Irvine, Irvine, CA 92697 USA
[4]Versatope Therapeutics, Inc., 110 Canal Street, Lowell, MA 01852 USA
[5]Lawrence Livermore National Laboratory, Livermore, CA 94550 USA
[6]Cornell Institute of Biotechnology, Cornell University, Ithaca, NY 14853 USA

Supplementary Note 1

The two most effective SNAPs in terms of biotin-GFP binding, namely eMA-IgAPβ and Lpp-OmpA-eMA, were evaluated over a range of conditions to identify parameters that affected GFP docking levels. A preliminary test of different cultivation variables (e.g., growth temperature, culture density at time of induction, inducer level, plasmid backbone, etc.), revealed that the density of the culture at the time of receptor induction had the greatest impact on the levels of biotin-GFP loading, with higher induction densities (Abs_{600} ≈ 1.8) resulting in SNAP-OMVs that captured the most antigen (**Supplementary Fig. 2a**). The Lpp-OmpA-eMA construct showed the highest biotin-GFP binding levels under the conditions tested. However, expression of this SNAP was detrimental to the host cells based on the observation that the final culture densities hardly changed, and in some

cases even decreased, from the densities at the time of induction, which was not the case for IgAP-eMA (**Supplementary Fig. 2b**). Given the different biogenesis pathways of the IgAP autotransporter versus the Lpp-OmpA β-barrel outer membrane protein, we suspected that the host cell toxicity associated with Lpp-OmpA might result from inducer levels that were too strong. In support of this notion, when Lpp-OmpA-eMA constructs were induced with ~50-times less inducer (0.27 mM vs. 13.3 mM L-arabinose), the post-induction cell growth was markedly improved, with Lpp-OmpA-eMA-expressing cells reaching a final density on par with that of cells expressing IgAP-eMA (**Supplementary Fig. 2c**). Importantly, the Lpp-OmpA-eMA SNAP-OMVs isolated from these healthier host cells captured significantly more biotin-GFP compared to IgAP-eMA SNAP-OMVs.

To determine whether these effects were specific to the choice of plasmid, we evaluated an alternative plasmid for expression of both SNAPs. Specifically, we re-cloned the IgAP-eMA and Lpp-OmpA-eMA constructs into the L-rhamnose-inducible plasmid, pTrham, which is known to afford tighter expression control compared to pBAD vectors and can help to overcome the deleterious saturation of membrane and secretory protein biogenesis pathways [1, 2]. As was observed with pBAD24, cells expressing IgAP-eMA from pTrham reached similar final densities regardless of the inducer levels, while growth of cells expressing Lpp-OmpA-eMA from pTrham decreased with increasing inducer levels (**Supplementary Fig. 2d**). Despite these differences in growth, IgAP-eMA and Lpp-OmpA-eMA SNAP-OMVs derived from cultures that were induced with 2 mM L-rhamnose each bound equivalent amounts of biotin-GFP (**Supplementary Fig. 2d**). Interestingly, increasing the amount of L-rhamnose yielded IgAP-eMA SNAP-OMVs that captured 2-3 times more biotin-GFP whereas decreasing the amount of L-rhamnose yielded Lpp-OmpA SNAP-OMVs that bound 4-5 times more biotin-GFP, consistent with the contrasting effects of inducer on the post-induction growth of cells expressing these constructs. Overall, the engineered Lpp-OmpA-eMA receptor expressed from pTrham plasmid using 0.5 mM L-rhamnose was the strongest performer in terms of biotin-GFP binding (**Supplementary Fig. 2d and e**); hence, we chose this plasmid/inducer combination for all further studies.

Supplementary Table 1. Plasmids used in this study

Name	Description	Reference
pET24a(+)-Cm	*E. coli* expression plasmid derived from pET-24a(+) but with Cm^R resistance marker; Cm^R	Lab stock
pET24-GFP	Encodes FACS-optimized GFPmut2 variant with C-terminal 6xHis tag in pET-24a(+)-Cm; Cm^R	This study
pET21d-Sx	Encodes SIMPLEx components MBP at the N-terminus and ApoAI* at the C-terminus with multicloning site for insertion of POIs between MBP and ApoAI*; Amp^R	[3]
pET21-Sx-Cm-MOMP	Encodes Cm-MOMP in pET21d-Sx; Amp^R	This study
pCM189	Yeast expression plasmid with tetracycline-regulated promoter; Amp^R	[4]
pCM-GFP	Encodes *S. cerevisae* codon-optimized GFP with a C-terminal 6xHis tag in pCM189; Amp^R	This study
pTrc99S-ssDsbA-CRM197^{4xDQNAT}	Encodes DsbA signal peptide fused in-frame with CRM197 followed by a 4x tandemly repeated DQNAT glycosylation tag in pTrc99S; Amp^R	[5]
pGAB2	Encodes *F. tularensis* SchuS4 O-PS biosynthesis pathway; Tet^R	[6]
pMAF10-PglB	Encodes *Campylobacter jejuni* PglB oligosaccharyltransferase in pMAF10; Tmp^R	[7]
pIVEX2.4d	Plasmid for *E. coli* cell-based and cell-free expression with a strong T7 promoter; Amp^R	[8]
pIVEX-Sx-CtE-MOMP	Encodes SIMPLEx fusion MBP-CtE-MOMP-ApoAI* in pIVEX2.4d; Amp^R	This study
pET45-rCm-MOMP	Encodes Cm-MOMP without its native signal peptide in pET-45b(+); Amp^R	[9]
pClyA-GFP	Encodes ClyA-GFPmut2 fusion in pBAD18-Cm; Cm^R	[10]
pBAD24-ClyA-eMA	Encodes ClyA-c-Myc-eMA-FLAG fusion in pBAD24; Amp^R	This study
pBAD24-Lpp-OmpA-eMA	Encodes Lpp-OmpA-c-Myc-eMA-FLAG fusion in pBAD24; Amp^R	This study
pBAD24-Intimin-eMA	Encodes Int-c-Myc-eMA-FLAG fusion in pBAD24; Amp^R	This study
pBAD24-eMA-Hbpβ	Encodes spPelB-FLAG-eMA-c-Myc-HBPβ fusion in pBAD24; Amp^R	This study
pBAD24-eMA-Ag43β	Encodes spPelB-FLAG-eMA-c-Myc-Ag43β fusion in pBAD24; Amp^R	This study
pBAD24-eMA-IgAPβ	Encodes spPelB-FLAG-eMA-c-Myc-IgAPβ fusion in pBAD24; Amp^R	This study
pBAD24-eMA-AIDA-Iβ	Encodes spPelB-FLAG-eMA-AIDA-Iβ fusion in pBAD24; Amp^R	This study

pTrham	*E. coli* expression vector containing L-rhamnose inducible promoter rhaBAD; Amp^R	Amid Biosciences
pTrham-Lpp-OmpA-eMA	Encodes Lpp-OmpA-c-Myc-eMA-FLAG fusion in pTrham; Amp^R	This study
pTrham-eMA-IgAPβ	Encodes ssPelB-FLAG-eMA-c-Myc-IgAPβ fusion in pTrham; Amp^R	This study
pTrham-Lpp-OmpA-SA^{S25H}	Encodes Lpp-OmpA-c-Myc-mSA^{S25H}-FLAG fusion in pTrham; Amp^R	This study
pTrham-Lpp-OmpA-SA	Encodes Lpp-OmpA-myc-SA-FLAG fusion in pTrham; Amp^R	This study
pTrham-Lpp-OmpA-RA	Encodes Lpp-OmpA-myc-RA-FLAG fusion in pTrham; Amp^R	This study

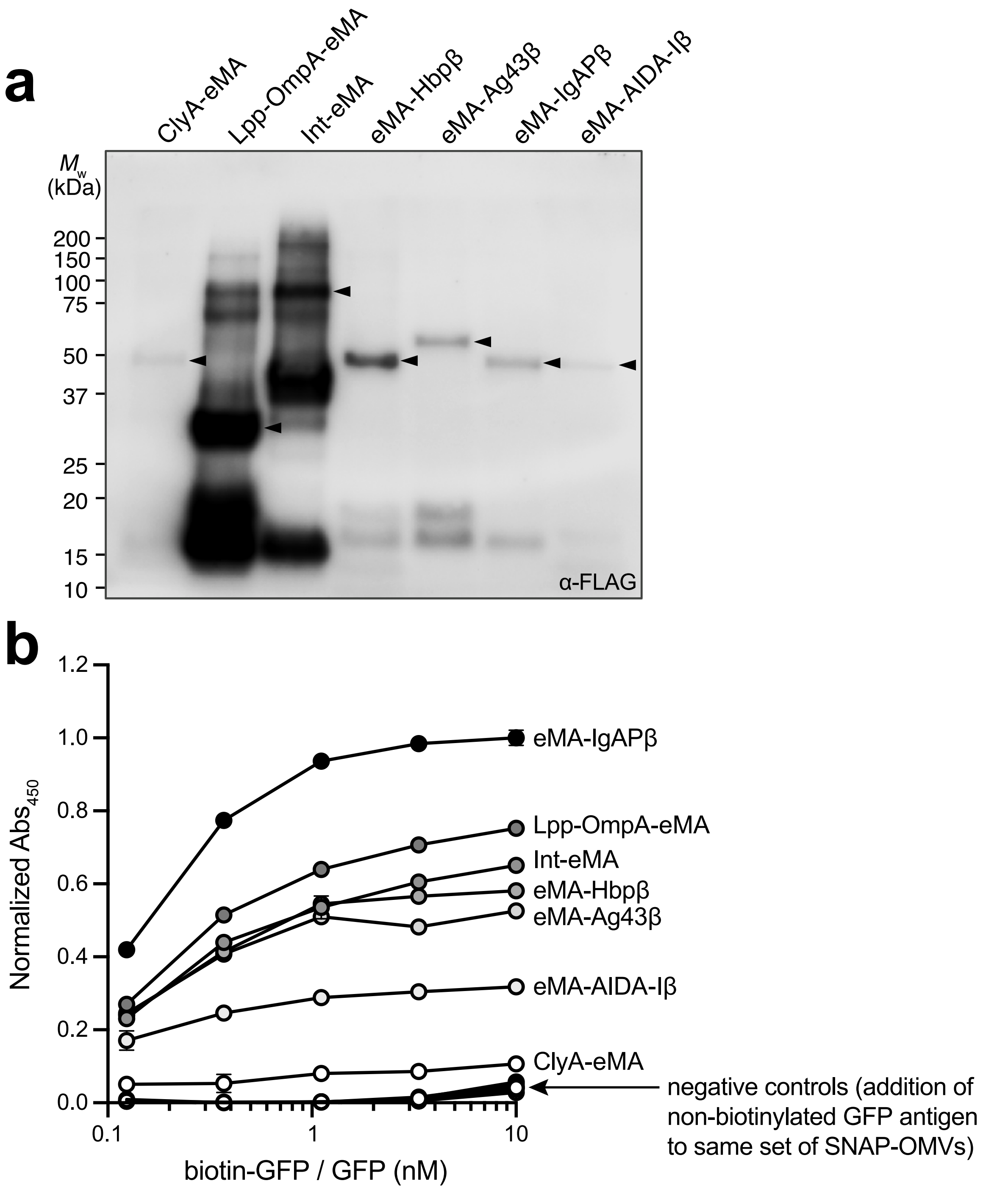

Supplementary Figure 1. Expression and antigen-binding activity of engineered SNAPs. (a) Immunoblot analysis of OMV fractions isolated from hypervesiculating *E. coli* strain KPM404 Δ*nlpI* expressing each of the different SNAPs from plasmid pBAD24. An equivalent amount of SNAP-OMVs as determined by total protein assay was loaded in each lane. Blot was probed with anti-FLAG antibody (α-FLAG) to detect FLAG epitope (DYKDDDDK) located at the N- or C-terminus of each construct. Expected location of full-length SNAP fusion proteins are denoted by black arrows. Molecular weight (M_w) ladder is indicated at left. (b) Binding of biotin-GFP to each of the different SNAP-OMVs as indicated. Binding activity was determined by ELISA in which biotin-binding SNAP-OMVs were immobilized on plates and subjected to varying amounts of biotin-GFP, after which plates were extensively washed prior to detection of bound biotin-GFP using anti-polyhistidine antibody to detect C-terminal 6xHis tag on GFP. Controls were performed by treating the same set of SNAP-OMVs with unmodfied GFP in place of biotin-GFP. All data were normalized to the maximum signal correpsonding to the eMA-IgAPβ construct in the presence of 10 nM biotin-GFP. Data are the mean ± SD with n = 2 biologically independent experiments. Source data are provided as a Source Data file.

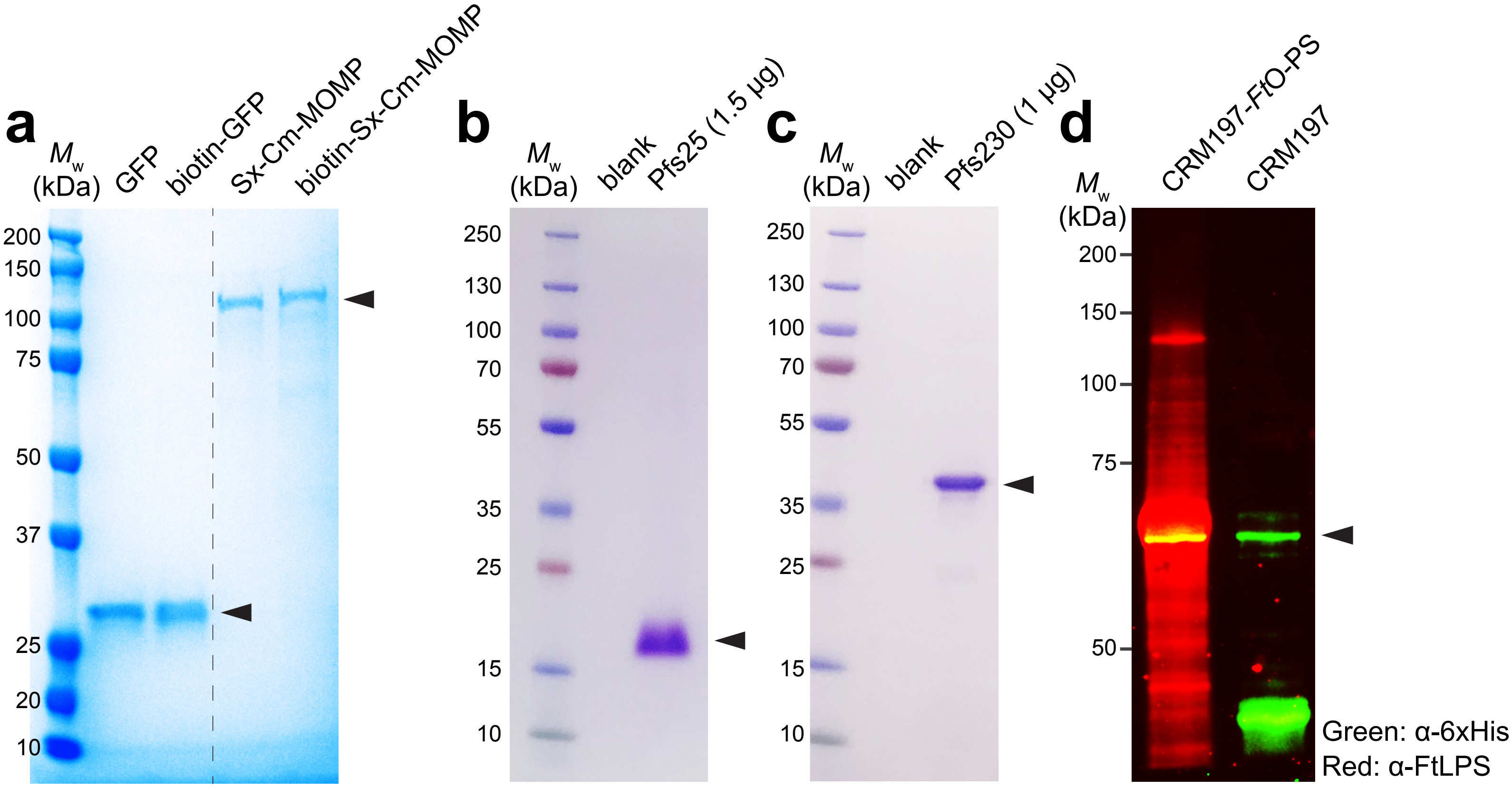

Supplementary Figure 2. Expression and purification of biotinylated protein antigens. Coomassie blue-stained SDS-PAGE gels of: (a) purified GFP and Sx-Cm-MOMP as well as their biotinylated counterparts; (b) purified Pfs25; and (c) purified Pfs230 (amino acids 443-731). GFP and Sx-Cm-MOMP were both produced using *E. coli* BL21(DE3), which yielded ~100 mg/L of GFP and ~5 mg/L of Sx-Cm-MOMP. Note that GFP was purified by nickel only, whereas SIMPLEx-MOMP was purified by Ni-NTA resin followed by amylose. Pfs25 and Pfs230 were produced by Syngene using a baculovirus expression system involving Sf9 cells and P2 virus, which yielded ~25 mg/L of each using Ni-NTA resin. (d) Immunoblot analysis of CRM197 with four tandemly repeated DQNAT glycosylation motifs purified from *E. coli* CLM24 with (left lane) or without (right lane) plasmid DNA encoding the *Ft*O-PS biosynthetic machinery. Glyconjugate yields were typically 2-3 mg/L. Blots were probed with anti-polyhistidine antibody (α6xHis; green signal) to detect the CRM197 carrier protein or FB11 (α FtO-PS; red signal) to detect the *Ft*O-PS glycan. Image shows merge of α6x-His and α*Ft*O-PS signals. High molecular weight laddering for red signal is characteristic of variable chain length O-PS polymers that are seen in native *Ft*LPS as well as in glycoconjugates derived from engineered *E. coli* (Stark et al. 2021 *Sci Adv*). All images are representative of at least three biological replicates. Molecular weight (M_w) markers are shown at the left of each image and expected sizes of full-length proteins are indicated by arrowheads. Source data are provided as a Source Data file.

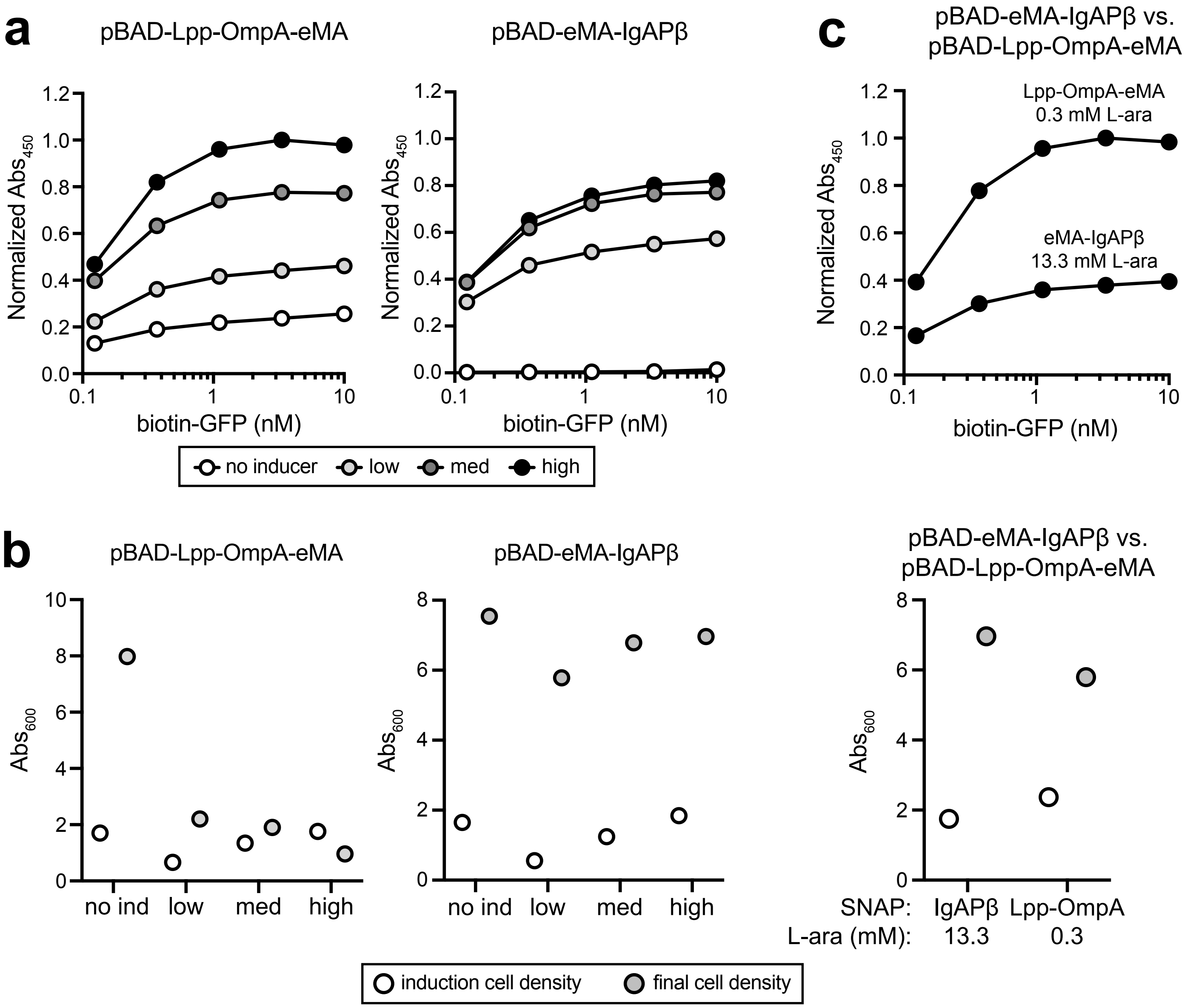

Supplementary Figure 3. Optimization of biotin-GFP docking on eMA-IgAPβ and Lpp-OmpA-eMA SNAPs expressed from pBAD24. (a) Binding of biotin-GFP to SNAP-OMVs isolated from hypervesiculating *E. coli* strain KPM404 Δ*nlpI* expressing Lpp-OmpA-eMA or eMA-IgAPβ from plasmid pBAD24 and induced at low (Abs_{600} ~0.6), medium (Abs_{600} ~1.2), or high (Abs_{600} ~1.8) culture density. Data in both graphs were normalized to the maximum binding signal corresponding to Lpp-OmpA-eMA SNARE-OMVs (high induction case) in the presence of 3.3 nM biotin-GFP. (b) Cell growth for same cultures in (a) where cell density was measured at time of induction (white bars) and just prior to harvesting SNAP-OMVs (gray bars). (c) Biotin-GFP binding and cell growth as in (a) and (b) but with 50-fold lower L-arabinose (L-ara) inducer for cells expressing Lpp-OmpA-eMA. Binding data were normalized to the maximum binding signal corresponding to Lpp-OmpA-eMA SNAP-OMVs in the presence of 3.3 nM biotin-GFP. Binding activity in all panels was determined by ELISA in which SNAP-OMVs were immobilized on plates and subjected to varying amounts of biotin-GFP, after which plates were extensively washed prior to detection with anti-polyhistidine antibody to detect C-terminal 6xHis tag on GFP. Data in (a) and (b) are the mean ± SD with n = 2 biologically independent experiments. Source data are provided as a Source Data file.

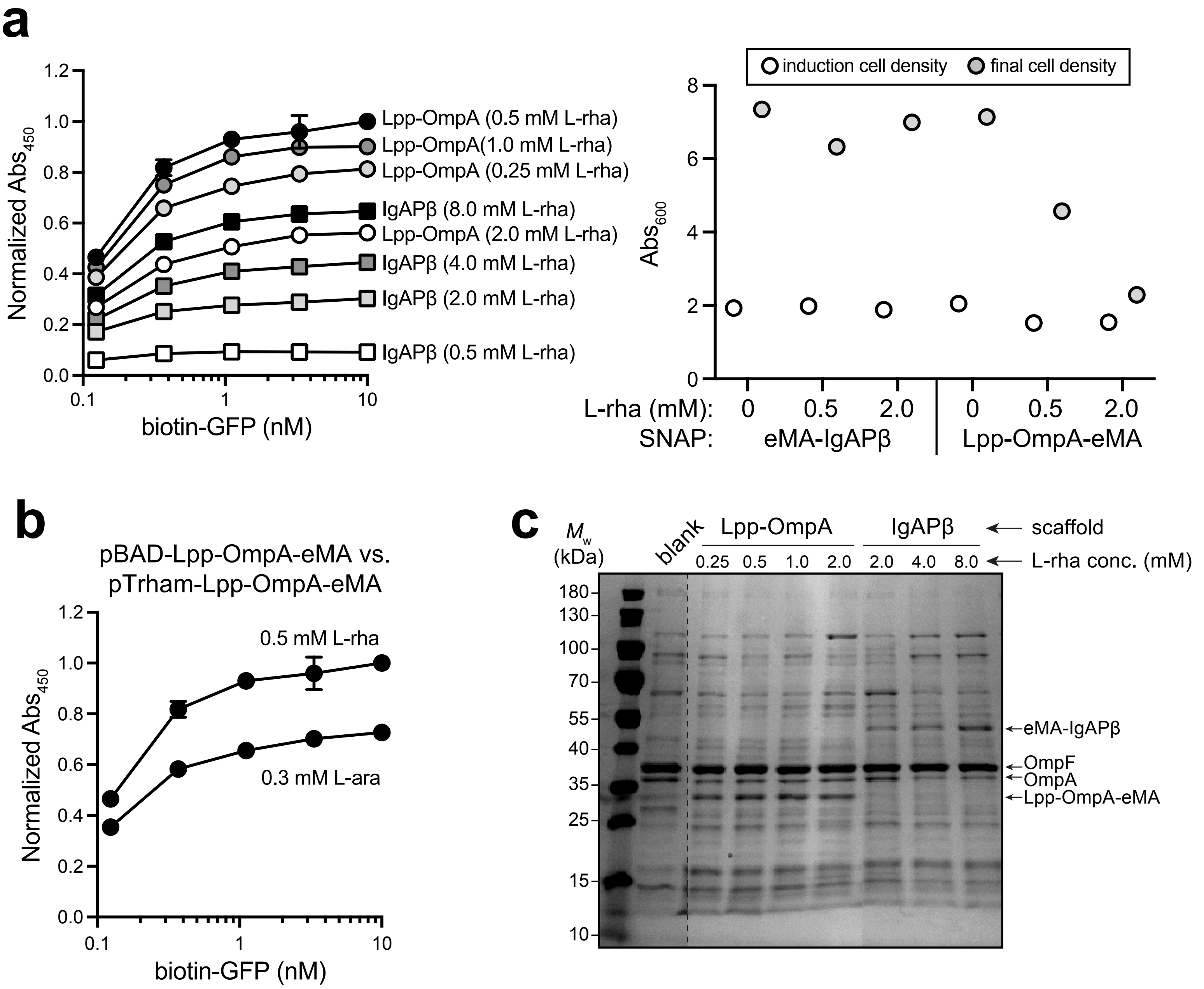

Supplementary Figure 4. Optimization of biotin-GFP docking on eMA-IgAPβ and Lpp-OmpA-eMA SNAPs expressed from pTrham. (a) (left) Biotin-GFP binding for SNAP-OMVs isolated from hypervesiculating *E. coli* strain KPM404 Δ*nlpI* expressing Lpp-OmpA-eMA or eMA-IgAPβ from plasmid pTrham in the presence of different amounts of L-rhamnose (L-rha) as indicated. (right) Cell growth for a subset of the cultures in panel at left where cell density (Abs_{600}) was measured at time of induction (white bars) and just prior to harvesting SNAP-OMVs (gray bars). Data are the mean ± SD with n = 2 biologically independent experiments. (b) Comparison of biotin-GFP binding for Lpp-OmpA-eMA expressed from pBAD24 versus pTrham with inducer amounts as indicated. Data in (a) and (b) were normalized to the maximum binding signal corresponding to the Lpp-OmpA-eMA construct expressed from pTrham in the presence of 0.5 mM L-rha. Binding activity in all panels was determined by ELISA in which SNAP-OMVs were immobilized on plates and subjected to varying amounts of biotin-GFP, after which plates were extensively washed prior to detection with anti-polyhistidine antibody to detect C-terminal 6xHis tag on GFP. Data are the mean ± SD with n = 2 biologically independent experiments. (c) Commassie blue-stained SDS-PAGE gel of blank OMVs or SNAP-OMVs isolated from cells containing either pTrham construct in the presence of different amounts of L-rha as indicated. Bands correspnding to SNAPs and the prevalent outer membrane proteins OmpA and OmpF are labeled. Molecular weight (M_w) ladder is indicated at left. Dashed line indicates splice of the same gel image to remove unused lanes. Blot representative of 2 independently repeated experiments with similar results. Source data are provided as a Source Data file.

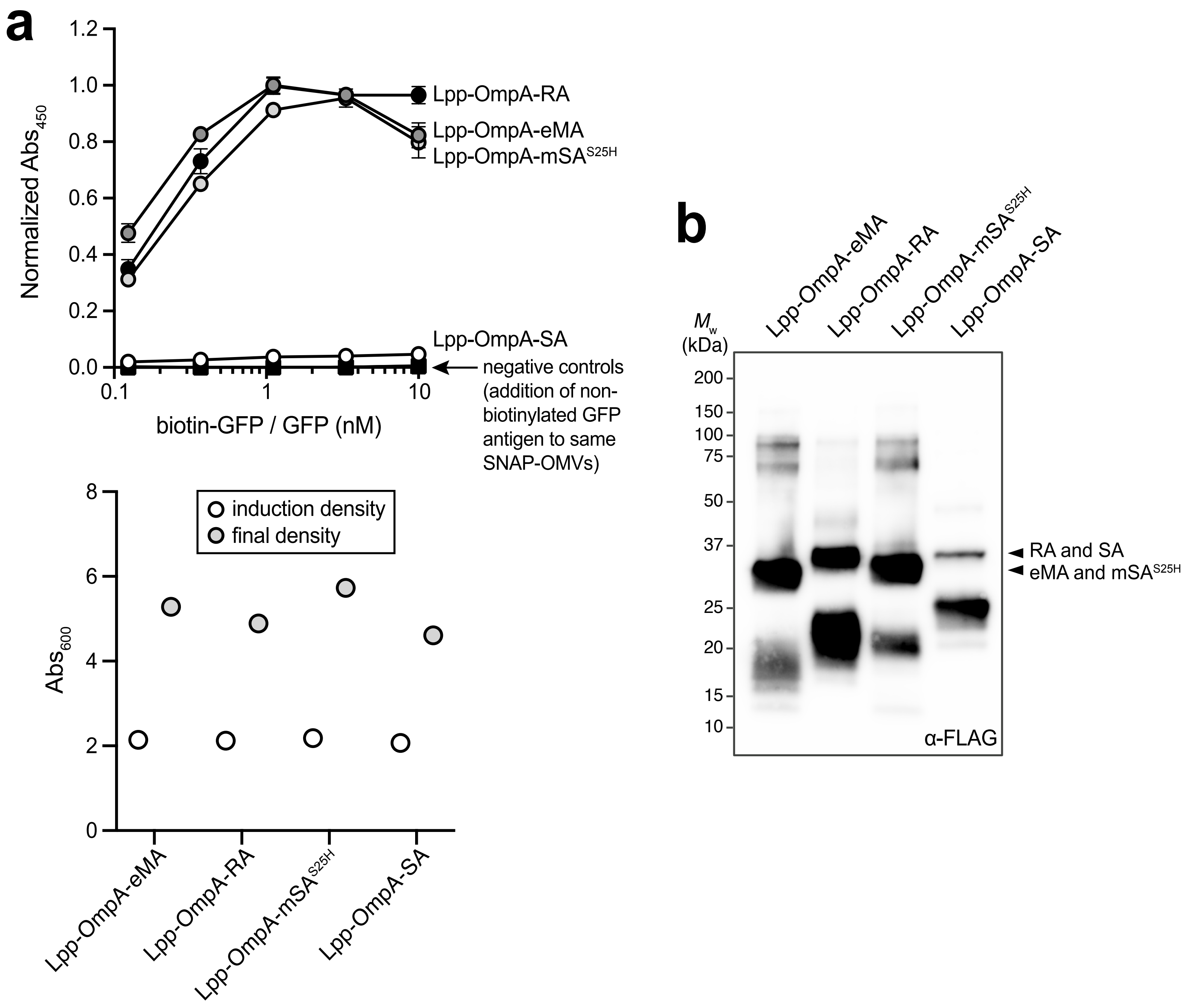

Supplementary Figure 5. Expression and antigen-binding activity of SNAPs with alternative biotin-binding modules. (a) (left panel) Binding of biotin-GFP to each of the different SNAP-OMVs as indicated. Binding activity was determined by ELISA in which biotin-binding SNAP-OMVs were immobilized on plates and subjected to varying amounts of biotin-GFP, after which plates were extensively washed prior to detection of bound biotin-GFP using anti-polyhistidine antibody to detect C-terminal 6xHis tag on GFP. Controls were performed by treating the same set of SNAP-OMVs with unmodfied GFP in place of biotin-GFP. All data were normalized to the maximum signal correpsonding to the Lpp-OmpA-eMA construct in the presence of 1 nM biotin-GFP. Data are the mean ± SD with n = 2 biologically independent experiments. (right panel) Cell growth for same cultures in (a) where cell density was measured at time of induction (white bars) and just prior to harvesting SNAP-OMVs (gray bars). (b) Immunoblot analysis of OMV fractions isolated from hypervesiculating *E. coli* strain KPM404 Δ*nlpI* expressing each of the different SNAPs from plasmid pBAD24. An equivalent amount of SNAP-OMVs as determined by total protein assay was loaded in each lane. Blot was probed with anti-FLAG antibody (α-FLAG) to detect FLAG epitope (DYKDDDDK) located at C-terminus of each construct. Expected location of full-length SNAP fusion proteins are denoted by black arrows. Molecular weight (M_w) ladder is indicated at left. Source data are provided as a Source Data file.

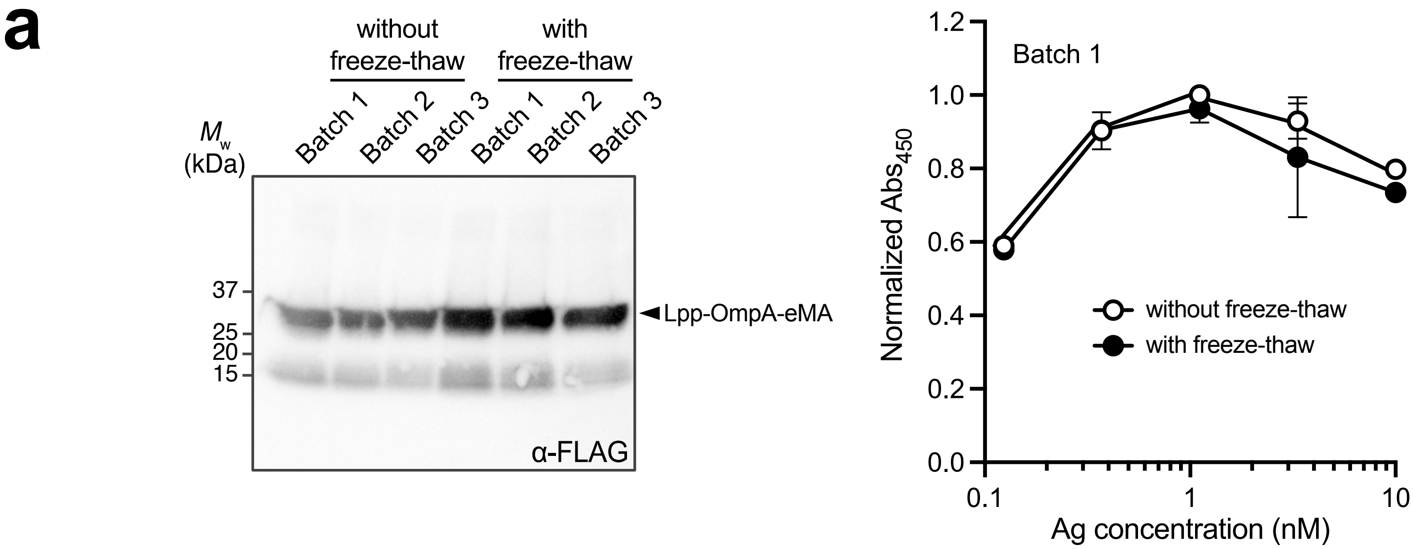

b

Sample	Z-average
SNAP-OMVs	131.2 ± 1.93 nm
SNAP-OMVs + GFP	130.2 ± 1.19 nm
SNAP-OMVs + biotin-GFP	131.5 ± 1.98 nm

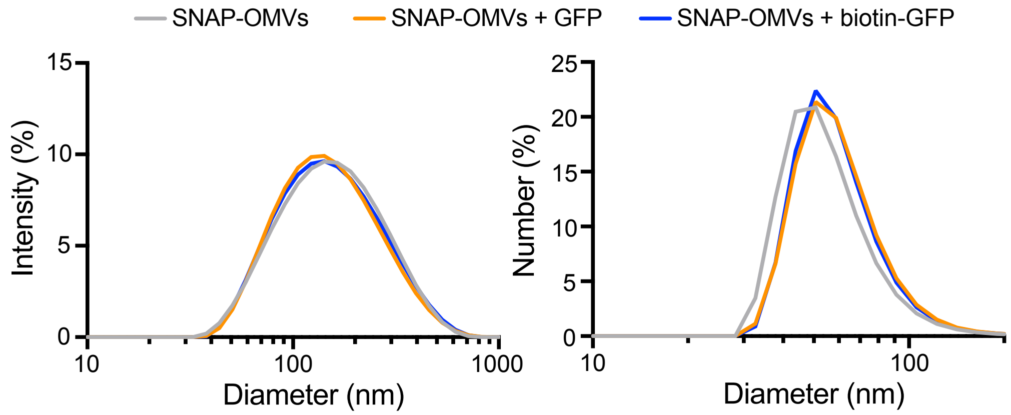

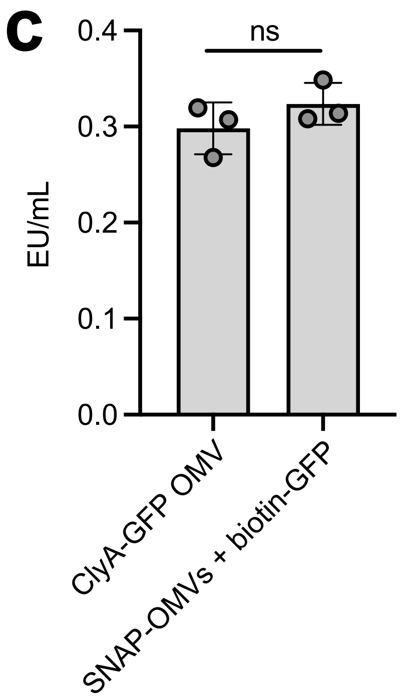

Supplementary Figure 6. Additional characterization of SNAP-OMVs. (a) Batch-to-batch comparision of Lpp-OmpA-eMA expression and activity. (top panel) Immunoblot analysis of OMV fractions isolated from three independent batches of hypervesiculating *E. coli* strain KPM404 Δ*nlpI* expressing Lpp-OmpA-eMA SNAP from plasmid pBAD24. OMV fractions were either loaded directly (without freeze-thaw) or subjected to freezing at -80°C then thawing (with freeze-thaw) prior to loading in gel. An equivalent amount of SNAP-OMVs as determined by total protein assay was loaded in each lane. Blot was probed with anti-FLAG antibody (α-FLAG) to detect FLAG epitope (DYKDDDDK) located at the C-terminus of each construct. Expected location of full-length Lpp-OmpA-eMA is denoted by black arrow. Molecular weight (M_w) ladder is indicated at left. (bottom panel) Representative dose-response curve generated by loading biotin-GFP on SNAP-OMVs prepared in Batch 1 and assayed as in Fig. 2a of main manuscript. Data were normalized to the maximum binding signal corresponding to Lpp-OmpA-eMA SNAP-OMVs in the presence of 3.3 nM biotin-GFP. Data are the mean ± SD with n = 2 biologically independent experiments. (b) Dynamic light scattering (DLS) analysis of SNAP-OMVs with and without GFP/biotin-GFP as indicated. Graph at left depicts intensity average; graph at right depicts number average. (c) Bacterial endotoxin test (BET) of Lpp-OmpA-eMA SNAP-OMVs containing biotin-GFP or ClyA-GFP OMVs assayed using Limulus Amebocyte Lysate (LAL) assay. Data are the mean ± SD with n = 3 biologically independent experiments. Statistical significance was determined by two-tailed t test with Welch's correction (ns – not significant). Actual p value = 0.2783. Source data are provided as a Source Data file.

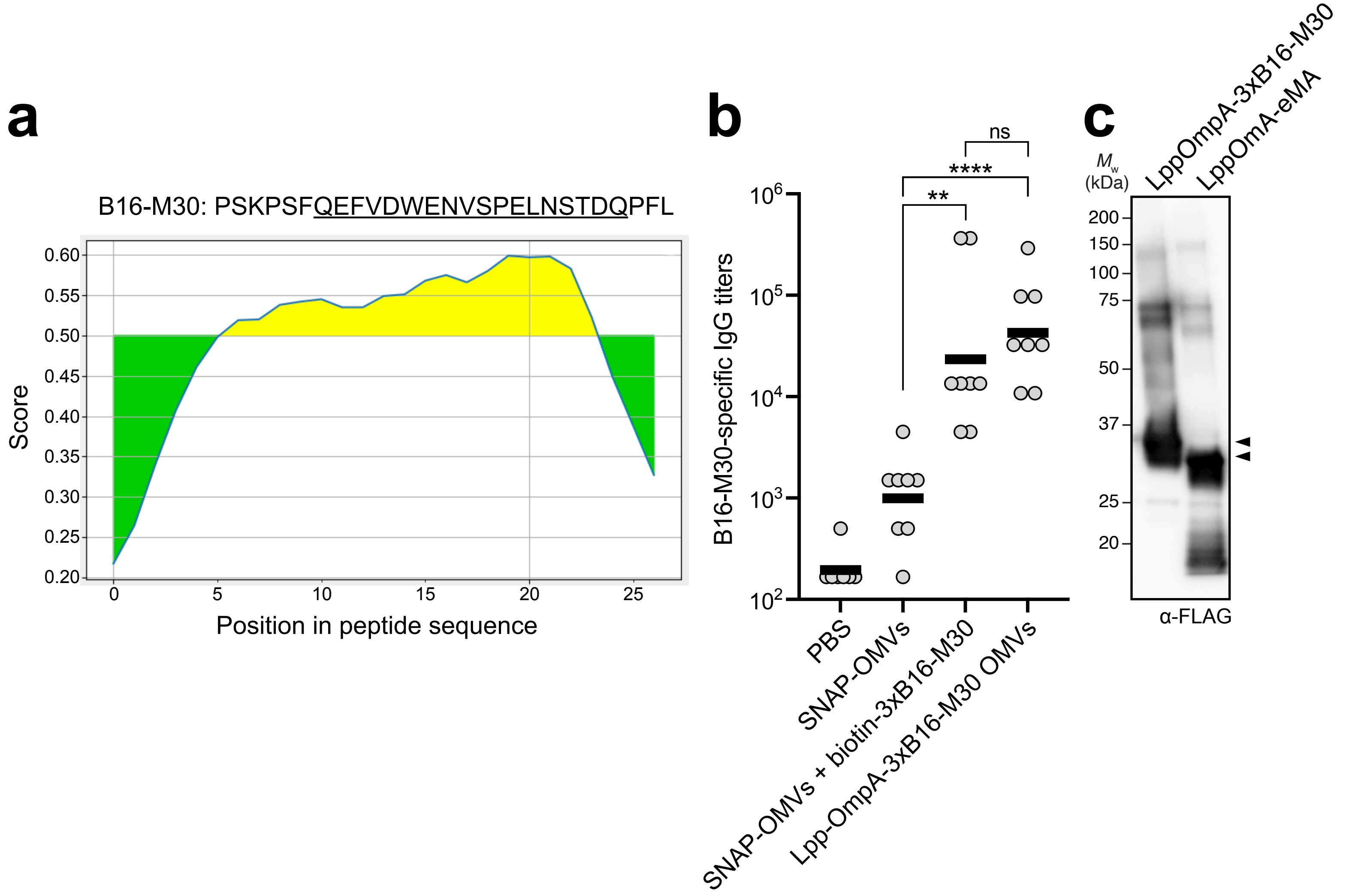

Supplementary Figure 7. SNAP-OMVs elicit IgG antibodies to linear peptide epitopes. (a) B-cell epitope prediction in B16-M30 peptide using the BepiPred-2.0 server, a sequential B-cell epitope predictor (Jespersen et al. 2017 *Nucleic Acids Res*). BepiPred-2.0 predicts B-cell epitopes from protein sequence, using a Random Forest algorithm trained on epitopes and non-epitope amino acids determined from crystal structures. A sequential prediction smoothing is performed afterwards. The residues with scores above the threshold (default value is 0.5) are predicted to be part of an epitope and colored in yellow. Underlined residues between Q7 and Q24 represent putative B-cell epitope. (b) Mean peptide-specific IgG titers (horizontal black lines) in endpoint (day 56) serum of individual mice in each group (gray circles). Four groups of BALB/c mice, eight mice per group, were immunized s.c. with the following: PBS, blank SNAP-OMVs, SNAP-OMVs mixed with 20 pmol biotinylated 3xB16-M30 peptide, and OMVs displaying an Lpp-OmpA-3xB16-M30 construct. All OMVs were isolated from KPM404 Δ*nlpI* cells with the appropriate expression plasmid. Mice received prime injections containing an equivalent amount of OMVs (20 μg total protein) on day 0 and were boosted on day 21 and 42 with the same doses. Antibody titering was performed by ELISA with single-copy B16-M30 peptide as immobilized antigen. Statistical significance was determined by two-tailed *t* test with Welch's correction (**, $p < 0.01$, ****, $p < 0.0001$; ns – not significant). Actual *p* values from left-to-right: $p = 0.0010$, $p < 0.0001$, and $p = 0.4335$. (c) Immunoblot analysis of OMV fractions isolated from hypervesiculating *E. coli* strain KPM404 Δ*nlpI* expressing either Lpp-OmpA-3xB16-M30 or Lpp-OmpA-eMA from plasmid pBAD24. An equivalent amount of OMVs as determined by total protein assay was loaded in each lane. Blot was probed with anti-FLAG antibody (α-FLAG) to detect FLAG epitope (DYKDDDDK) located at C-terminus of each construct. Expected location of full-length Lpp-OmpA-3xB16-M30 and Lpp-OmpA-eMA fusion proteins are denoted by black arrows. Molecular weight (M_w) ladder is indicated at left. Source data are provided as a Source Data file.

Supplementary References

1. Giacalone, M.J. et al. Toxic protein expression in *Escherichia coli* using a rhamnose-based tightly regulated and tunable promoter system. *Biotechniques* **40**, 355-364 (2006).
2. Hjelm, A. et al. Tailoring *Escherichia coli* for the l-rhamnose PBAD promoter-based production of membrane and secretory proteins. *ACS Synth Biol* **6**, 985-994 (2017).
3. Mizrachi, D. et al. A water-soluble DsbB variant that catalyzes disulfide-bond formation *in vivo*. *Nat Chem Biol* **13**, 1022-1028 (2017).
4. Garí, E., Piedrafita, L., Aldea, M. & Herrero, E. A set of vectors with a tetracycline-regulatable promoter system for modulated gene expression in *Saccharomyces cerevisiae*. *Yeast* **13**, 837-848 (1997).
5. Stark, J.C. et al. On-demand biomanufacturing of protective conjugate vaccines. *Sci Adv* **7**, eabe9444 (2021).
6. Cuccui, J. et al. Exploitation of bacterial *N*-linked glycosylation to develop a novel recombinant glycoconjugate vaccine against *Francisella tularensis*. *Open Biol* **3**, 130002 (2013).
7. Feldman, M.F. et al. Engineering *N*-linked protein glycosylation with diverse O antigen lipopolysaccharide structures in *Escherichia coli*. *Proc Natl Acad Sci U S A* **102**, 3016-3021 (2005).
8. Roge, J. & Betton, J.M. Use of pIVEX plasmids for protein overproduction in *Escherichia coli*. *Microb Cell Fact* **4**, 18 (2005).
9. Sun, G., Pal, S., Weiland, J., Peterson, E.M. & de la Maza, L.M. Protection against an intranasal challenge by vaccines formulated with native and recombinant preparations of the *Chlamydia trachomatis* major outer membrane protein. *Vaccine* **27**, 5020-5025 (2009).
10. Kim, J.Y. et al. Engineered bacterial outer membrane vesicles with enhanced functionality. *J Mol Biol* **380**, 51-66 (2008).
