# Supplementary material for: A modular vaccine platform enabled by decoration of bacterial outer membrane vesicles with biotinylated antigens
Source: Nat Commun. 2023 Jan 28;14:464. doi: 10.1038/s41467-023-36101-2 (PMC9883832; doi:10.1038/s41467-023-36101-2)
Supplement: Supplementary file 2 — Reporting Summary [file 41467_2023_36101_MOESM2_ESM.pdf]

nature portfolio

Corresponding author(s): Matthew DeLisa

Last updated by author(s): Dec 29, 2022

Reporting Summary

Nature Portfolio wishes to improve the reproducibility of the work that we publish. This form provides structure for consistency and transparency in reporting. For further information on Nature Portfolio policies, see our Editorial Policies and the Editorial Policy Checklist.

Statistics

For all statistical analyses, confirm that the following items are present in the figure legend, table legend, main text, or Methods section.

n/a	Confirmed	
☐	☒	The exact sample size (*n*) for each experimental group/condition, given as a discrete number and unit of measurement
☐	☒	A statement on whether measurements were taken from distinct samples or whether the same sample was measured repeatedly
☐	☒	The statistical test(s) used AND whether they are one- or two-sided<br>*Only common tests should be described solely by name; describe more complex techniques in the Methods section.*
☒	☐	A description of all covariates tested
☒	☐	A description of any assumptions or corrections, such as tests of normality and adjustment for multiple comparisons
☐	☒	A full description of the statistical parameters including central tendency (e.g. means) or other basic estimates (e.g. regression coefficient) AND variation (e.g. standard deviation) or associated estimates of uncertainty (e.g. confidence intervals)
☐	☒	For null hypothesis testing, the test statistic (e.g. *F*, *t*, *r*) with confidence intervals, effect sizes, degrees of freedom and *P* value noted<br>*Give P values as exact values whenever suitable.*
☒	☐	For Bayesian analysis, information on the choice of priors and Markov chain Monte Carlo settings
☒	☐	For hierarchical and complex designs, identification of the appropriate level for tests and full reporting of outcomes
☒	☐	Estimates of effect sizes (e.g. Cohen's *d*, Pearson's *r*), indicating how they were calculated

Our web collection on statistics for biologists contains articles on many of the points above.

Software and code

Policy information about availability of computer code

Data collection	Image Lab 6.1 software (Bio-Rad) was used for collecting Western blot images.
Data analysis	Image Lab 6.1 software (Bio-Rad) was used for analyzing Western blots. Prism 9 for macOS (version 9.4.1) was used for generating all graphs and performing all statistical analysis described in the manuscript.

For manuscripts utilizing custom algorithms or software that are central to the research but not yet described in published literature, software must be made available to editors and reviewers. We strongly encourage code deposition in a community repository (e.g. GitHub). See the Nature Portfolio guidelines for submitting code & software for further information.

Data

Policy information about availability of data

All manuscripts must include a data availability statement. This statement should provide the following information, where applicable:

- Accession codes, unique identifiers, or web links for publicly available datasets
- A description of any restrictions on data availability
- For clinical datasets or third party data, please ensure that the statement adheres to our policy

All data generated or analyzed during this study are included in this article and its Supplementary Information/Source Data file that are provided with this paper.

Human research participants

Policy information about studies involving human research participants and Sex and Gender in Research.

Reporting on sex and gender	N/A
Population characteristics	N/A
Recruitment	N/A
Ethics oversight	N/A

Note that full information on the approval of the study protocol must also be provided in the manuscript.

Field-specific reporting

Please select the one below that is the best fit for your research. If you are not sure, read the appropriate sections before making your selection.

[x] Life sciences [ ] Behavioural & social sciences [ ] Ecological, evolutionary & environmental sciences

For a reference copy of the document with all sections, see nature.com/documents/nr-reporting-summary-flat.pdf

Life sciences study design

All studies must disclose on these points even when the disclosure is negative.

Sample size	For experiments other than those involving mice, sample sizes were not predetermined based on statistical methods, but were chosen according to the standards of the field (at least three independent biological replicates for each condition), which gave sufficient statistics for the effect sizes of interest. To justify the proposed number of animals, a power analysis was performed (2 sample, equal variance) with alpha = 0.05. The analysis indicated that for the studies to have a 90% chance of detection (beta = 0.1), 7 mice were necessary per study group, which is consistent with the literature, and is consistent with our research team's extensive work with OMV vaccines and their capacity to induce an immune response.
Data exclusions	No data was excluded from the analyses in this work.
Replication	To verify the reproducibility of results from experiments other than those involving mice, we performed three biological replicates of each. In every experiment presented, the results were found to be reproducible. For animal experiments, replication was achieved by using seven mice in each group.
Randomization	The non-animal experiments were not randomized. All samples were analyzed equally with no sub-sampling and thus there was no requirement for randomization. Likewise, all mice were analyzed equally with no sub-sampling and thus there was no requirement for randomization.
Blinding	For all experiments other than those involving animals, investigators were not blinded. Blinding during collection was not needed because conditions were well controlled. Blinding during analysis was not feasible as the differences between samples under different conditions were visually apparent in the collected data. Blinding is also not necessary because the results are quantitative and did not require subjective judgment or interpretation. Blinding is not typically used in the field. For animal experiments, investigators were not blinded. Blinding was not deemed necessary as all animal data was acquired indiscriminately by the experimenters following well-defined pre-established criteria.

Reporting for specific materials, systems and methods

We require information from authors about some types of materials, experimental systems and methods used in many studies. Here, indicate whether each material, system or method listed is relevant to your study. If you are not sure if a list item applies to your research, read the appropriate section before selecting a response.

Materials & experimental systems

n/a	Involved in the study	
[ ]	[x]	Antibodies
[ ]	[x]	Eukaryotic cell lines
[x]	[ ]	Palaeontology and archaeology
[ ]	[x]	Animals and other organisms
[x]	[ ]	Clinical data
[x]	[ ]	Dual use research of concern

Methods

n/a	Involved in the study	
[x]	[ ]	ChIP-seq
[x]	[ ]	Flow cytometry
[x]	[ ]	MRI-based neuroimaging

Antibodies

Antibodies used	Avidin expression on OMVs was analyzed with horseradish peroxidase (HRP)-conjugated anti-c-Myc (Abcam; Cat # ab19312) or HRP-conjugated anti-DDDDK (Abcam; Cat # ab1162) antibodies that recognized c-Myc and FLAG epitope tags, respectively. Proteins and peptides bearing C-terminal 6xHis tags were detected with mouse anti-6xHis antibody clone AD1.1.10 (BioRad; Cat # MCA1396GA) while detection of glycosylated CRM197-FtO-PS was with anti-F. tularensis LPS antibody clone FB11 (Invitrogen; Cat # MA1-21690) that is specific to FtLPS. For probing antigenicity of SIMPLEx constructs, Sx-Cm-MOMP and Sx-CtE-MOMP were detected by Western blot analysis with mAb MoPn-40 (1:1,000; produced in-house) or anti-CtE-MOMP (1:2,000; Novus Biologicals; Cat # NB100-66403) antibodies. For ELISA-based detection of antigens, the following additional antibodies were used: GD2 was detected with mouse anti-ganglioside GD2 antibody (1:1,000; Abcam; Cat # ab68456); LeY was detected with mouse anti-Lewis Y antibody clone H18A (1:1,000; Absolute Antibody; Cat # Ab00493-1.1); DNP was detected with goat anti-DNP (1:5,000; Bethyl Laboratories; Cat # A150-117A); and PC was detected with anti-phosphorylcholine antibody clone BH8 (1:250; MilliporeSigma; Cat # MABF2084). HRP-conjugated donkey anti-goat secondary was used as needed (1:5,000; Abcam; Cat # ab97110). For titering antibodies in immune sera, the following antibodies were used: HRP-conjugated goat anti-mouse IgG (1:10,000; Abcam Cat # ab6789); HRP-conjugated anti-mouse IgG1 (1:10,000; Abcam Cat # ab97240), and HRP-conjugated anti-mouse IgG2a (1:10,000; Abcam Cat # ab97245). Finally, HRP-conjugated goat anti-mouse (Abcam; Cat # ab6789) or IRDye 800CW-conjugated goat anti-mouse secondary antibodies (1:10,000; Li-Cor; Cat # 926-32210) were used as needed.
Validation	All antibodies used in this work were comprehensively validated for quality and performance (specificity, sensitivity, cross-reactivity) as discussed on the vendor websites . Detailed protocols for usage of all of these antibodies can also be found at the following websites:<br>1. horseradish peroxidase (HRP)-conjugated anti-c-Myc (Abcam; Cat # ab19312); https://www.abcam.com/hrp-c-myc-antibody-ab19312.html<br>2. HRP-conjugated anti-DDDDK (Abcam; Cat # ab1162); https://www.abcam.com/ddddk-tag-binds-to-flag-tag-sequence-antibody-ab1162.html<br>3. mouse anti-6xHis antibody clone AD1.1.10 (BioRad; Cat # MCA1396GA); https://www.bio-rad-antibodies.com/monoclonal/synthetic-peptide-histidine-tag-antibody-ad1-1-10-mca1396.html?f=purified&JSESSIONID_STERLING=4F7961A797DE20E67408D3E75AD06F4F.ecommerce2&evCntryLang=US-en&cntry=US&thirdPartyCookieEnabled=true<br>4. anti-F. tularensis LPS antibody clone FB11 (Invitrogen; Cat # MA1-21690); https://www.fishersci.com/shop/products/anti-francisella-tularensis-lps-clone-fb11/MA121690<br>5. HRP-conjugated goat anti-mouse (Abcam; Cat # ab6789); https://www.abcam.com/goat-mouse-igg-hl-hrp-ab6789.html<br>6. anti-CtE-MOMP (Novus Biologicals; Cat # NB100-66403); https://www.novusbio.com/products/chlamydia-trachomatis-momp-antibody-1297-143_nb100-66403<br>7. IRDye 800CW-conjugated goat anti-mouse secondary antibody (Li-Cor; Cat # 926-32210); https://www.licor.com/bio/reagents/irdye-800cw-goat-anti-mouse-igg-secondary-antibody<br>8. mouse anti-ganglioside GD2 antibody (Abcam; Cat # ab68456); https://www.abcam.com/ganglioside-gd2-antibody-14g2a-ab68456.html<br>9. mouse anti-Lewis Y antibody clone H18A (Absolute Antibody; Cat # Ab00493-1.1); https://absoluteantibody.com/product/anti-ley-h18a/<br>10. goat anti-DNP (Bethyl Laboratories; Cat # A150-117A); https://www.thermofisher.com/antibody/product/DNP-Antibody-Polyclonal/A150-117A<br>11. anti-phosphorylcholine antibody clone BH8 (MilliporeSigma; Cat # MABF2084); https://www.emdmillipore.com/US/en/product/Anti-Phosphorylcholine-Antibody-clone-BH8,MM_NF-MABF2084-200UL<br>12. HRP-conjugated donkey anti-goat secondary (Abcam; Cat # ab97110); https://www.abcam.com/donkey-goat-igg-hl-hrp-ab97110.html

Eukaryotic cell lines

Policy information about cell lines and Sex and Gender in Research

Cell line source(s)	Spodoptera frugiperda Sf9 cells were used to produce two of the antigens used in this study, namely Pfs25 and Pfs230. The cell lines were obtained and used by a commercial service provider, Syngene.
Authentication	Sf9 cells were authenticated by Syngene's in-house cell-line development service facility (https://www.syngeneintl.com/solutions/discovery-biology/cell-line-development-analysis/).
Mycoplasma contamination	Sf9 cells were routinely tested for sterility and Mycoplasma contamination at Syngene's in-house cell-line development service facility (https://www.syngeneintl.com/solutions/discovery-biology/cell-line-development-analysis/).
Commonly misidentified lines<br>(See ICLAC register)	N/A

Animals and other research organisms

Policy information about studies involving animals; ARRIVE guidelines recommended for reporting animal research, and Sex and Gender in Research

Laboratory animals	Wild-type BALB/c mice (7 mice per group); six weeks old. Mice were housed under the following environmental conditions to reduce stress: 14-hour light/10-hour dark cycle and temperature of ~70F with ~50% humidity.

Wild animals	No wild animals were used in the study.
Reporting on sex	Both male and female mice were used.
Field-collected samples	No field-collected samples were used in the study.
Ethics oversight	The protocol number for the animal trial was 2012-0132 and was approved by the Institutional Animal Care and Use Committee (IACUC) at Cornell University.

Note that full information on the approval of the study protocol must also be provided in the manuscript.
